# Review of nanotheranostics for molecular mechanisms underlying psychiatric disorders and commensurate nanotherapeutics for neuropsychiatry: The mind knockout

**DOI:** 10.7150/ntno.49619

**Published:** 2021-03-01

**Authors:** Rajiv Kumar, Bhupender S Chhikara, Kiran Gulia, Mitrabasu Chhillar

**Affiliations:** 1 NIET, National Institute of Medical Science, India.; 2 Department of Chemistry, Aditi Mahavidyalaya, University of Delhi. Delhi, 110039, India.; 3 Materials and Manufacturing, School of Engineering, University of Wolverhampton, England, TF2 9NN, UK.; 4 Institute of Nuclear Medicine and Allied Sciences (INMAS) Brig. S. K. Mazumdar Marg Delhi 110054, India.

**Keywords:** Psychiatric disorders, nanotheranostics, nanotherapeutics, neurodegeneration, pro-inflammatory cytokines, microglial dysfunction, and kynurenine pathway

## Abstract

Bio-neuronal led psychiatric abnormalities transpired by the loss of neuronal structure and function (neurodegeneration), pro-inflammatory cytokines, microglial dysfunction, altered neurotransmission, toxicants, serotonin deficiency, kynurenine pathway, and excessively produced neurotoxic substances. These uncontrolled happenings in the etiology of psychiatric disorders initiate further changes in neurotransmitter metabolism, pathologic microglial, cell activation, and impaired neuroplasticity. Inflammatory cytokines, the outcome of dysfunctional mitochondria, dysregulation of the immune system, and under stress functions of the brain are leading biochemical factors for depression and anxiety. Nanoscale drug delivery platforms, inexpensive diagnostics using nanomaterials, nano-scale imaging technologies, and ligand-conjugated nanocrystals used for elucidating the molecular mechanisms and foremost cellular communications liable for such disorders are highly capable features to study for efficient diagnosis and therapy of the mental illness. These theranostic tools made up of multifunctional nanomaterials have the potential for effective and accurate diagnosis, imaging of psychiatric disorders, and are at the forefront of leading technologies in nanotheranostics openings field as they can collectively and efficiently target the stimulated territories of the cerebellum (cells and tissues) through molecular-scale interactions with higher bioavailability, and bio-accessibility. Specifically, the nanoplatforms based neurological changes are playing a significant role in the diagnosis of psychiatric disorders and portraying the routes of functional restoration of mental disorders by newer imaging tools at nano-level in all directions. Because of these nanotherapeutic platforms, the molecules of nanomedicine can penetrate the Blood-Brain Barrier with an increased half-life of drug molecules. The discoveries in nanotheranostics and nanotherapeutics inbuilt unique multi-functionalities are providing the best multiplicities of novel nanotherapeutic potentialities with no toxicity concerns at the level of nano range.

## Introduction

Psychiatric disorders (neurodegenerative diseases) occur after a series of intriguing incidences such as inflammatory dysfunctions and increased neuroinflammation (peripheral and central nervous system) 1. These factors collectively activate microglia cells (immune and structural cells) to induce the release of pro-inflammatory cytokines interleukin (IL) -1β, (IL) -18, and tumor necrosis factor-alpha (TNF) -α, that initiates neuronal damages and trigger neuron death 2. These cells further released fragmented and dysfunctional mitochondria into the brain tissue milieu. Activated microglial cells catalyze astrocyte activity and push them to release glutamate for initiating the kynurenine pathway, very much toxic activity of the central nervous system (CNS), and further influence dopaminergic, serotonergic, and glutamatergic signaling pathways 3. Nano-scale imaging technologies and ligand-conjugated nanocrystals can diagnose molecular mechanisms that govern the cellular communication of mental illness. It makes the diagnosis by sensing the surface of phase-sensitive neuronal as part of a cerebellum tissue, and also further, the neurotransmitter diffusion in the brain assist the imaging of multifaceted neural activities quantitatively and qualitatively under the electrical and chemical stimulations. In absence of proper diagnostics tools, and improper scientific knowledge, mental illnesses (depression, anxiety disorders, addictions, personality disorders, schizophrenia, eating disorders and addictive behaviors) remain undiagnosed, and because of it, patients cannot address such diseases at the time of initiation that leads to devastating consequences (individual suffering, shattered relationships, and health complications) which may emerge any time 4. Therefore, nanotheranostics and nanotherapeutics are the best tools available to treat psychiatric disorders (depression, hopelessness, mood disorders, anxiety disorders, schizophrenia, post-traumatic stress disorder, addictive behaviors, eating disorders, and personality disorders). These include: developing nanodevices for (the diagnosis of the disease), nanocarriers (better drug delivery), and nanorobots (to commensurate nanotherapeutics as per the demand of neurophysiological correlations with depression and other mental illnesses) 5.

The attitudes and behaviors concerning one's illness are products of deduced practices to commemorate, and therefore, it can be affected by cognitive dysfunctions. To understand the mental states of other, it is necessary to replay upon "to see ourselves as others see us". This improves the abilities to know the mental statuses (opinions, beliefs, awareness, and intentions) of others. Therefore, the correct outlook to moody alteration in oneself depends on the abilities to reveal upon personality as a prospective, and that is, the priority of the theory of mind. Insight has to think of as a relational concept that serves as the light of understanding into something i.e. understanding illness, pathological nature traits, specific symptoms, current syndrome, and social difficulties, etc. 6. Therefore, the nanomedicine with inbuilt and self-protected abilities to prevent themselves from degradation during surface modification into distinct molecules during the action, so it can be used for the sophisticated detection of signals from the mental state, neural, physiological, and muscle. Neurology, psychology, and psychiatry mechanisms are the key components that take part in the various functions of the physiology and are highly responsible for disorders of the brain. Elementary motors and sensory processes are the key features of adequate assessment of “brain function” that govern cognition and behavior 7. These symptoms are (1) interlinked by psychological processes, (2) investigated by psychological upset, (3) initiated secondary psychological reaction, and (4) psychological manifestations that (5) affect the brain.

Finally, there are many pieces of evidence for elucidating the role of neuroinflammation in various psychiatric illnesses as abnormal neurotransmission, serotonin deficiency, because of the changes in neurotransmitter metabolism, pathologic microglial cell activation, impaired neuroplasticity, and structural and functional brain changes responsible for cognition and emotional behavior 9. Psychoses clinically expressed as cognitive conditions, (delusions, delirium, dementia, schizophrenia, and hallucinations) of the brain. Inflammatory cytokines and increased production of neurotoxic substances for postulating the link and culprit among the key causes for enhancing disease progression 10. As per an emerging theory, the main devil is stress, which plays a key role in initiating the deregulation of the immune system in psychiatric diseases, alongside genetic, epigenetic, and environmental factors. Illnesses such as schizophrenia, autism, depression, and other mood disorders linked with inflammation of the brain, however, the exact underlying mechanisms of these kinds of relationships are specific to each illness. The outcome of any dysregulation of the immune system in the brain might lead to the origin and occurrence of depression and anxiety. The analysis of the imbalance in the cytokines' concentration, the inflammatory component, verify the psychiatric symptoms and inflammation 11, which can initiate stress and anxiety to activate depression. To encounter such negative effect and paraphernalia of these factors on the physiology of the nerve cells, there is a need to develop such well-equipped nanocarriers with specific abilities and multifunctional capabilities (transport for hydrophobic entities, active and passive targeting, size exclusion, potential to detect diseases (as imaging tools), deliver treatments without toxicity 12. They can easily circulate through blood vessels, uniformly distributed, penetrate the cell membranes, with the ability to hold macromolecules and not let decompose it outside and inside the cell. These nanoplatforms derived from multifunctional nanomaterials of specific properties can serve as diagnostic tools, monitoring sites, for the treatment and prevention of diseases (inflammatory conditions, neurodegenerative and psychiatric diseases, diabetes, and infectious diseases) 13,14. The negative effects of the dysregulated cytokine networks may start the pathogenesis of affective disorders (depression and anxiety). Therefore, further investigation of the causes responsible for inflammation in psychiatry disorders, and how these correlate themselves with the known alterations in the mechanism of each disease, may further create new opportunities for the development of a more satisfactory treatment/intervention, method and it will have particular effectiveness to prevent diseases. In our impression, it is an attempt to develop new strategies towards the proper understanding of the applications of nanomaterials in cognitive behavior/cognitive sciences 15.

## Insights of molecular mechanisms underlying Psychiatric Disorders

Toxicants enter cellular, molecular, and inflammatory pathways to start the initiation of processes for the origin of diseases. These foreign objects damage the brain's cellular process. These destructions in the functioning of neurotransmission processes and protein networking cycles propagate the pathogenesis of neuropsychiatric diseases. To encounter these undesirable difficulties in the cell's physiology, the cytokines, an interface regulator for interactions and communications between cells, stimulate the neuronal defense fortresses of immune cells to secrete anti-inflammatory cytokines for healing the prolonged pain 16. A variety of specific cytokines and their neutralizing antibodies recommended for clinical use may be a potent remedy to cure stroke, Alzheimer's disease, amyotrophic lateral sclerosis, autoimmune diseases, and wound healing. Some organs cannot stop the entry of toxicants in the absence of a compact Blood-Brain Barrier (BBB) and thus enhance the probability of neurodegeneration 17. The excessive extracellular cerebral excretion process of glutamatergic cytokine interferes in excitotoxicity induced by it in neuropathology. The other side of defense systems, microglia are there as the key component during such reaction mechanisms of the neuron, the activation of these components of neurons during neuroinflammation, initiate the release of mediators (nitric oxide, chemokines, and proinflammatory cytokines). It is crystal clear that the reactive oxygen species/ reactive nitrogen species (ROS/RNS), free radical nitric oxide (NO∙), and its derivative the peroxynitrite (ONOO^-^), an influential oxidant, capable to damage the biological molecules are among the main damaging constituents that exist in brain functioning. As in the necrosis process, tumor necrosis factor-alpha (TNF) -α medicate cross-linkages between neurons and astrocytes for further engagements 18.

**Figure 1:**
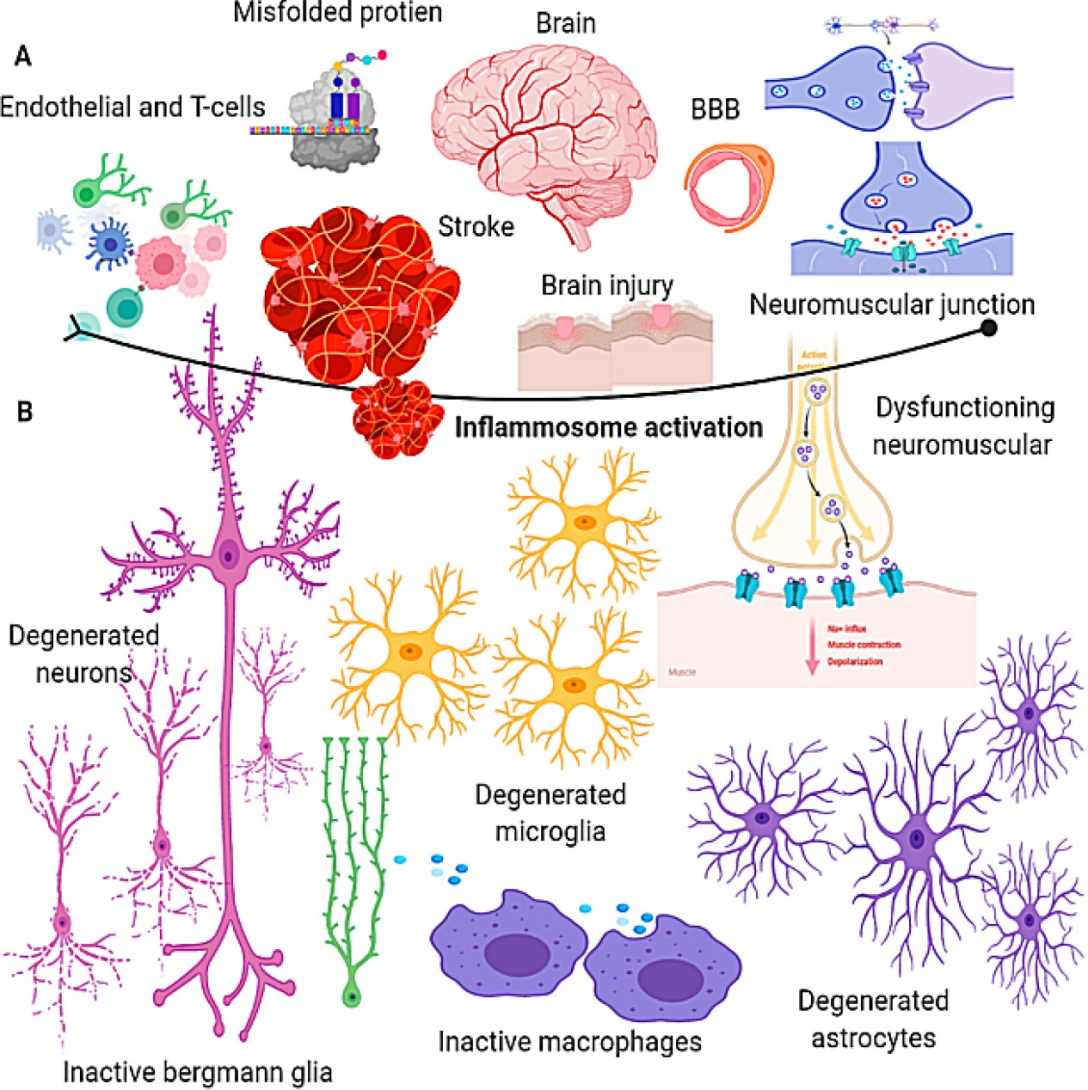
Insights of molecular mechanisms underlying psychiatric disorders. Related steps of Inflammation activation by underling the key roles of endothelial, misfolded proteins, neuromuscular junction, inflammatory activation, endothelial cells, T-cells, and dysfunction of neuromuscular. Defected neuron cells: degenerated microglia, degenerated astrocytes, inactive macrophages, and Bergmann glia, and degenerated neurons. (A) Brain, brain injury, BBB, neuromuscular junction, misfolded proteins, and endothelial cells. (B) Degenerated neurons, microglia, astrocytes, bergmann gila, and inactive macrophages.

***a)**** Impact of oxidative stress on the neuron:* Lake of sufficient oxygen (O_2_) in the cell causes reduced production of ATP. Such dysfunctions force neurons to reduce their activities, and as a result, the neurodegenerative processes get started. But the interrelated mechanism is not still clear and how it happened, also not yet discovered. The free radicals and non-radical derivatives are there for signal sensitization in the brain to deal with oxidative stress whenever redox signaling goes awry (negative functionality). The ribonucleic acid (RNA) oxidation is an underappreciated cause of oxidative stress 19. Undiscovered facts, machinery, and mechanisms going on in the neurons are among the main causes to dictate neuronal susceptibility, the dynamics of oxidative stress, and particularly based on neural identity, **Figure 1.** To disclose the further distinct biochemical proceedings, the nanotheranostics must urgently employ for the observations of oxidative stress in the brain and disease 20.

Tight homeostatic control over the production of ROS in normal cells, containing neurons, is essential for smooth cell regulation. There is a need to detoxify ROS, and biological antioxidants (glutathione, α-tocopherol (a vitamin E), carotenoids, and ascorbic acid), because these react with most of the oxidants available at the spot. Besides, the antioxidant enzymes (catalase and glutathione peroxidase) detoxify hydrogen peroxides (H_2_O_2_) by converting it to (O_2_) and water (H_2_O). Oxidative stress, the deleterious condition, occurs whenever ROS intensities go beyond the antioxidant volume of a cell. As the neurons take part chemically via neurotransmitters for chemical transmission, and neuromodulation (glutamate, gamma-aminobutyric acid, acetylcholine, and peptide transmitters) 21. An imbalance between over-production and/or under-detoxification of oxidative/nitrosative stress) leads to hypoxia and hypoglycemia, which are the principal causes among those who are responsible for oxidative stress 22,23. A lot of modifications in the structural units of lipids, nucleic acids, proteins, and modulating the function of macromolecules exist in cellular transformation. After the initiation of oxidative stress, which leads it into either dysfunction or these molecules loose their specified activities. As a result, a lot of molecular and cellular mechanisms and their interrelated factors de-regularize soon. These pathological states initiate the degeneration process within the dopaminergic neurons. The high intrinsic oxidative stress, low adenosine triphosphate assembly, dysregulation, dysfunction, and damages of the mitochondrial, and extraordinary inflammatory reactions influenced the repair the damages and deficient deoxyribonucleic acid, propagate low calcium-buffering ability, over activation of glutamate receptors, influence the lipid turnover, influence protein refolding, DNA base excision, and repair 24. The ROS attack nucleic acids in several ways, initiate the breaking in the strand of DNA-protein cross-links and may change purine, pyrimidine bases resulting in DNA mutations. During these cellular processes, the neurons looked for high energy, and therefore, there is a high demand for ATP production and the same has already reduced because of dysfunction of the mitochondrion, and finally, this causes a negative impact on neuron functionality 25. Uncontrolled generation of the ROS do damages to the cellular components, environment, and organelles and initiate apoptosis, and narcosis 26,27. Neuronal networking promotes self-defense through lymphatic tissue and glial cells, and starting of the circulation of the spinal fluid and white blood cells in the vessels during the removal of metabolic waste yields isolated toxins, and dead cells.

**Figure 2:**
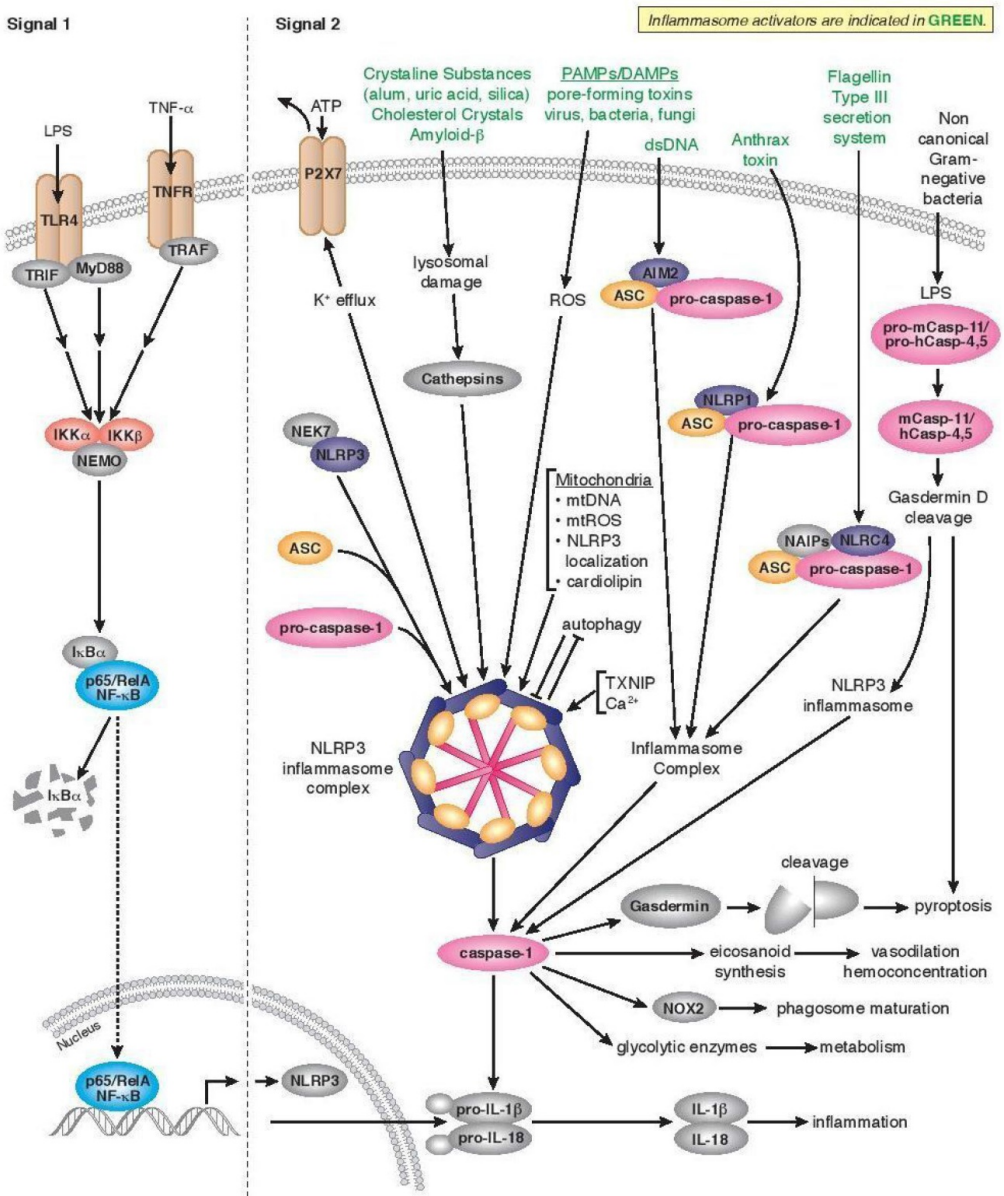
Neuroinflammation and neurodegenerative diseases by using nanomedicine induced inflammasome as autoimmunity nanotherapeutics. Transformations at molecular and cellular levels start the inflammasomes which start acting as an intracellular sensor between microbial (host) and foreign pathogens (unwanted guests) by starting signaling for unwanted happenings followed by the discharging the inflammatory cytokines interleukin (IL)-1β and (IL)-18, in self-defense. Adapted with permission from [www.cellsignal.com] and the same is acknowledged.

***b)****Inflammasomes in neuroinflammation and neurodegenerative diseases:* Aggregated host proteins (amyloid-β, α-synuclein, and prions), deposits abnormally within the cell, and near the territories of the cells that initiate neuroinflammation and neurodegeneration 28. Such transformations at molecular and cellular levels initiate the inflammasomes which start acting as an intracellular sensor between microbial (host) and foreign pathogens (unwanted guests) by starting signaling for unwanted happenings followed by the discharge of the inflammatory cytokines interleukin (IL)-1β and (IL)-18, in self-defense. In the meantime, other cellular processes like pyroptosis ensued to catalyze the anti-inflammatory chemicals 1. The mechanisms of inflammasome activation in the brain, controlled by immune cells (microglia). Further, astrocytes, neurons, and myeloid cells collectively activate inflammasomes to enhance self-defense. This discussion highlights the remedial options available to treat neuroinflammation and neurodegenerative diseases by using nanomedicine induced inflammasome as autoimmunity nanotherapeutics 29 and illustrated in **Figure 2.**

The cytotoxic consequences and existence of aggregated misfolded proteins are liable for chronic neuroinflammation and psychiatric disorders 30 Errors in protein folding, initiate negativity to disturb the normal routines, and the functionalization processes of the cells and cellular processes 31. Microglia has a natural ability to sense the existence of such irregular happenings within proteins and transformation proceedings and promote selected cell mechanisms to encounter them and throw toxins/harmful substances out of it. Such self-cleaning abilities composed of the biochemical processes help in regulating neuroinflammation and as a result, the stages of neurodegenerative disease vanished 32 Thus, the harmful stimuli, phagocytizing debris, and apoptotic neurons are easily detectable by astrocytes and microglia 18. Soon, nanotheranostics may have such kinds of abilities as inbuilt or a much more advanced version of it could innovate soon. These newer strategies will be available and highly capable of immunomodulatory functioning for monitoring synaptic homeostasis and facilitating clearance of apoptotic cells as ended by astrocytes and microglia 33. Therefore, the need for nanotherapeutics induced inflammasomes is the state-of-the-art approaches to lead the discovery of better drug formulation for novel treatment of neuroinflammation and neurodegenerative diseases 34.

***c)****Cellular and molecular neuroinflammatory pathways and kynurenine pathway induce neurodegeneration:* The mechanism of neuroinflammation governed by neurovascular units (microglial, and glial cells, neuron cells, and endothelial cells) perform as a platform to coordinate with pro- and anti-inflammatory mechanisms 35. The brain and peripheral cells have to check the opening mechanism of inflammatory as interceded by cytokines, chemokines, and reactive oxygen species. Further, these can easily initiate local or CNS inflammation 36.

The mechanism of neurovascular unit dysfunction affected by neuroinflammation and mishappenings as infections. So, the process of neuroinflammation is likely to be a normalization effort in response to any offense affecting the host-defense mechanism 3. The activation of microglia, T-lymphocyte infiltration, and overproduction of inflammatory cytokines are the first line of offense associated with the restoration of normal structural and functional mechanisms of the brain 37. The neutralization process of an infection initiated by the discharge of molecular mediators such as inflammatory cytokines, and prostaglandin E2, caused by nitric oxide, reactive oxygen, and nitrogen species and at once act to foil neurodegeneration 38. Thus, cellular and molecular mediators most likely assist in pathological processes responsible for a disease progression. This has practically proved by injecting proteinaceous infectious particles, followed by the detection of the symptoms of mental illnesses. But a total number of 1,000 trillion viruses and over 100,000,000,000,000 bacteria have detected in the human gut or as per ration 150 times more bacterial DNA than human DNA in a human body 39.

**Figure 3:**
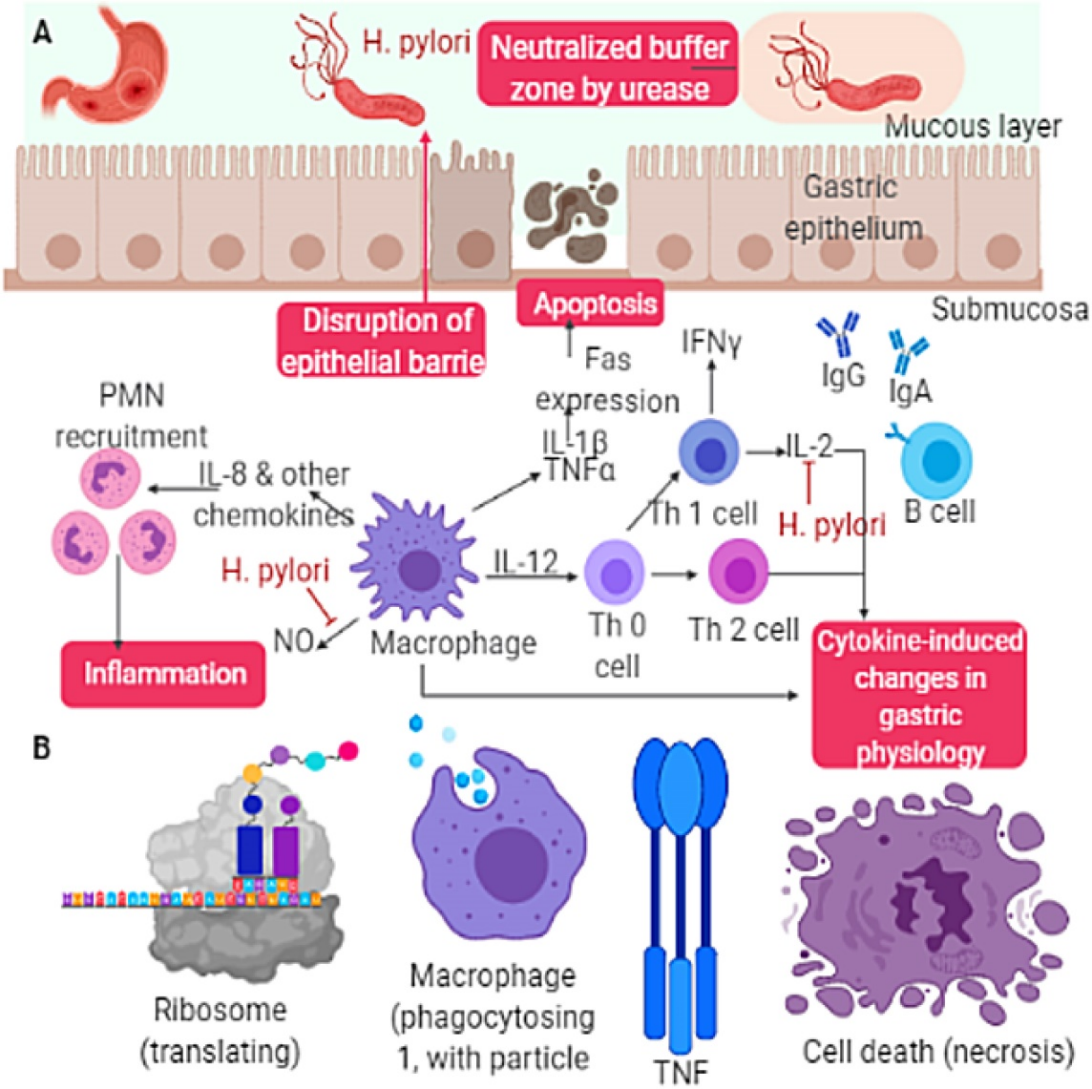
Pathological processes leading to disease progression and the role of phenotypes in neuroinflammation. The oxidative injury occurred induced by an oxidative burst in microglia, amplified by mitochondrial damage. The activation signal mechanism activates cellular phenotypes (astrocytes, microglia, and peripheral immune cells), and these signaling mechanisms for initiating agents against cytokines, misfolded protein, infectious agents. (A) H. pylori activities on the surface of gastric epithelium, apoptosis cycle (B) Components of apoptosis, ribosome (translating), macrophage, TNF, and necrosis.

Viruses (herpes, cytomegalovirus, and HIV) normally existed in the brains of diseased persons as having the protozoa Toxoplasma *gondii.* It bases the pathways of these mechanisms on different activation signals and mediators. These activation signals activate cellular phenotypes (astrocytes, microglia, and peripheral immune cells), and these signaling mechanisms further initiate controlling of the cytokines secretion, misfolded protein mechanism, and infection 40, and presented in **Figure 3.** The mechanisms of the activation of the phenotypes for responding towards the signals received from the multifaceted neuron-microglial of the CNS, and how they secrete different responding factors to alter the signal of forces, are the key mechanisms to highlight 41. What does it happen between the cellular phenotypes and the inflammatory mediators? and what will the alteration events of the molecular and cellular routes, neuronal signals, and receptors mechanism? The mechanisms are holding the front. By disinterring, such nameless mechanisms of cellular activities may further assist in the discovery of novel and potential nanotheranostics and related strategies. Major transformation and cellular mechanisms, such as the association of molecular mechanisms with underlying mechanism dysfunction neurovascular unit procedure, are among the key pinpoints to learn 42. When all these mechanisms take place on the active sites are still unclear. How oxidative injury occurred induced by an oxidative burst in microglia, amplified by mitochondrial damage and iron liberation within lesions, are the impending challenges and upcoming soon-to-be 43.

**Figure 4:**
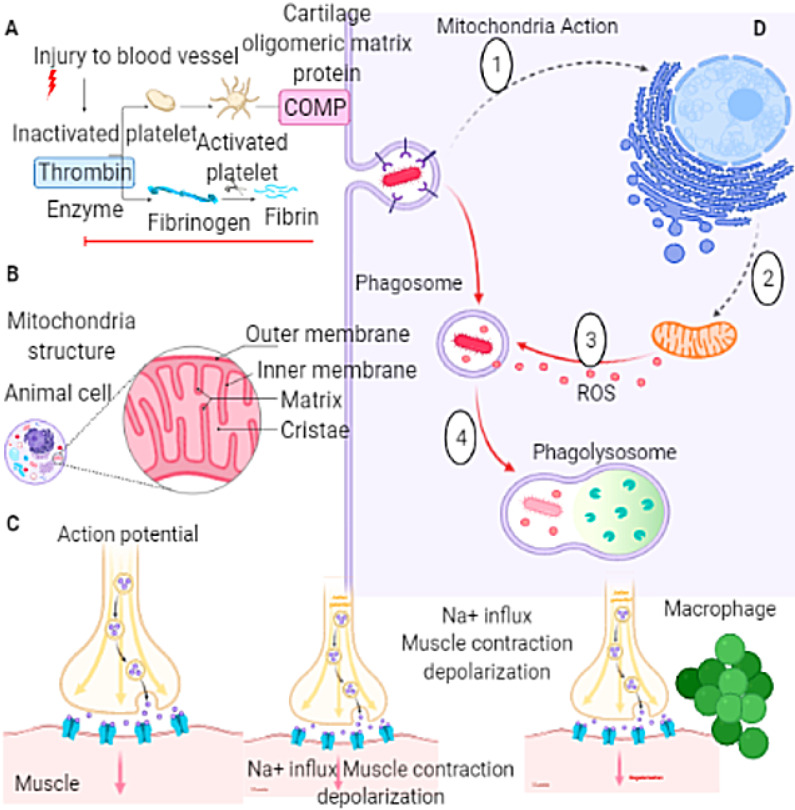
The mechanisms of mitochondrial dysfunction, reactive astrocytes, and neuronal cell death. The mechanism of dysfunction of mitochondria dysregulated responsible for the necrotic neuronal death. The processes of mental illness cause an assessment of the factors that involve in determining progressions of reported mild dysfunction roots psychiatric disorders. **(A)** Cycle of the injury to the blood vessels **(B)** Components of the mitochondria, **(C)** Sodium ion influx, muscle contraction depolarization,** (D)** Route of the action of the mitochondria.

***d)**** Mitochondrial dysfunction, reactive astrocytes, and neuronal cell death:* Concerning dynamics of mitochondria governs a lot of important mechanisms i.e. ROS production and sequestrations, ATP production, intermediate metabolism, neurotransmitter generation and degradations, apoptosis, and Ca^2+^ buffering 44. The mitochondria, itself, also undergoes several processes or phenomena i.e. mitochondrial fission, fusion, mitochondrial bioenergetics, and transport 24. Other key features of these cellular and molecular types of machinery are special neurons, astrocytes in the brain governed many functions of CNS i.e. glutamate, calcium, potassium ions (Ca^2+^, K^+^), tissue repair via angiogenesis and neurogenesis, energy storage, mitochondria biogenesis, defense against oxidative/nitrosative stress, and synaptic modulation 45. The disease-specific proteins (aggregated, β-amyloid, and α-synuclein), cleared by astrocytes through the large-conductance Ca^2+^-activated K+ channels 46. Upgraded nanotheranostics strategies will have to escort with these high profiling cellular processes governed by the astrocytes. Such innovations will play an important role in the innovation of a complete set of nanotherapeutics 47. Here, the most important process or mechanism occurs to fulfill the energy needs of the astrocytes. It gets completed and fulfilled by mitochondrial networking. Any perturbations in the astrocyte and mitochondrion energy supply chain reaction, immediately distressed neuron functioning and as a result, neuronal damage transpired (neurodegeneration), **Figure 4.** The chief causes associated with neurodegeneration are dysregulation of Ca^2+^ homeostasis and mitochondrial dysfunction 48. As proof of the pieces of evidence, as mitochondrion helped in cytosolic Ca^2+^ waves and global Ca^2+^ signaling and soon, thereafter, the mechanism of dysfunction of mitochondria dysregulated Ca^2+^ related mechanisms responsible for the necrotic neuronal death occurred just because of dysfunctional and dysregulation 49. Nanotheranostics may further explore such hidden and undiscovered aspects of these multifaceted mechanisms (electron transport mechanism and interrelated pathways, decreased ATP production at cellular level, decreased pH in the cellular environment, increased Ca^2+^ concentration in the cells, the discharge mechanism of glutamate, increased concentration of the arachidonic acid, gene alterations and activation leading to cytokine synthesis, synthesis of enzymes involved in free radical production, and accumulation of leukocytes) 50,51.

Identifying these critical functional and structural changes and their mechanisms of action responsible for cell death are still unknown. Shortly, only the nanotheranostics will be the best option for exploring newer diagnostic and imaging techniques to fulfill the existing needs 52. As, L-tryptophan (Trp), known as a watchdog of protein homeostasis, if it degrades, then it yields various active molecules of metabolites, necessary for metabolism and it classifies this pathway as kynurenine pathway. The same metabolites influenced cellular components and environment (the endocrine, hemopoietic, immune system, metabolism pathways, and neuronal networking), and ensued kynurenine pathway, a necessity for vital cellular functioning and routes 53. The multifactorial nature of the processes of mental illness causes an assessment of the factors that involve in determining progressions of reported mild dysfunction roots of psychiatric disorders 54. The developing strategies (nanotheranostics and nanotherapeutics) arrest, heal, and revert these progressions responsible for neuron death.

## Nanotheranostics induced molecular imaging, diagnosis neuropsychiatry: Exploring the mechanisms underlying inflammation, and neurodegeneration

**Figure 5:**
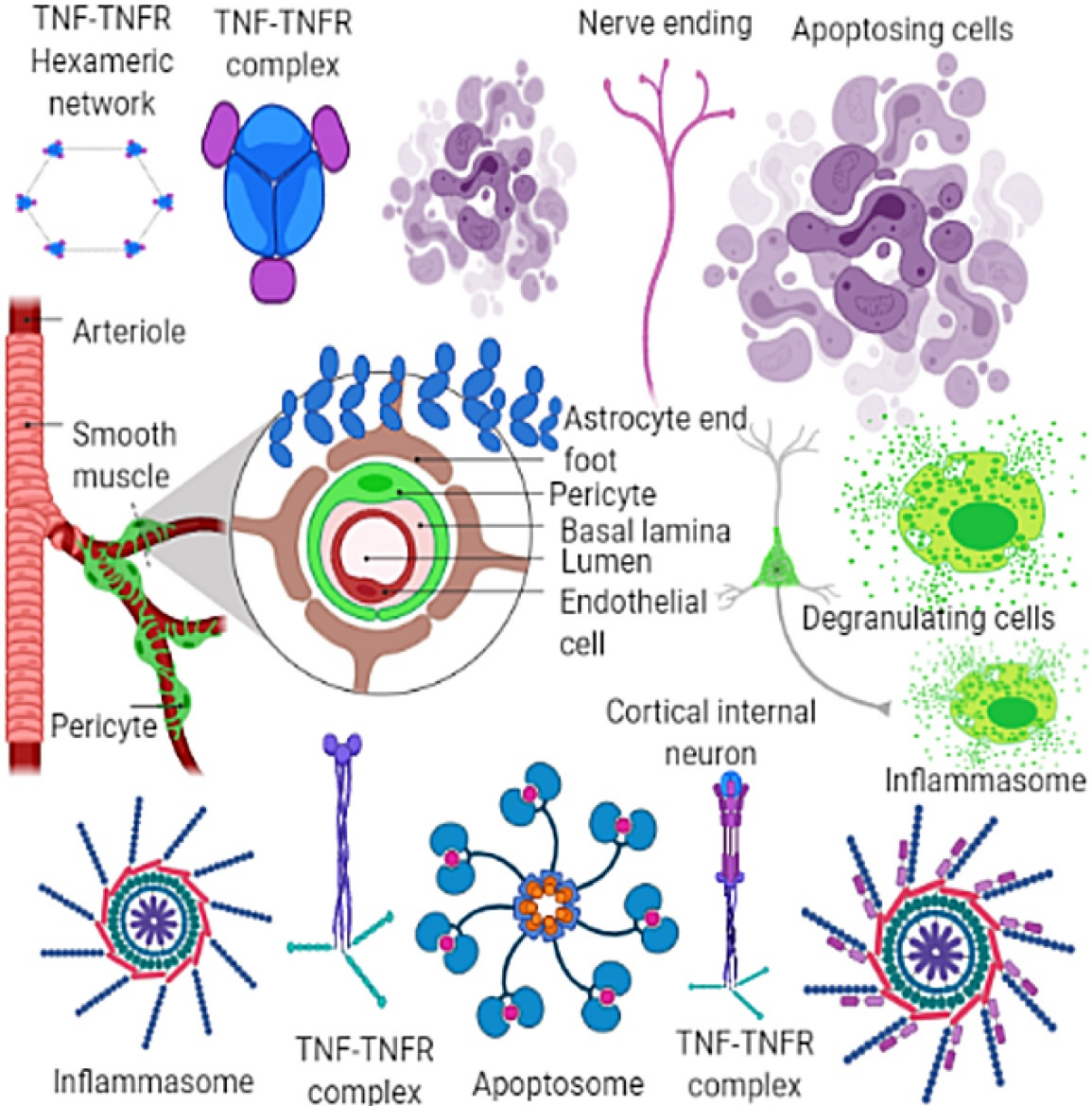
The neurobiological features, neurotransmitter pathways, structural abnormalities on neuroimaging, and genetic predispositions. Anorexia regulated possibilities of exploring the mechanisms underlying inflammation and neurodegeneration. Possibilities of detection by bioinformatics and modern genomic tools.

To distinguish between mental illnesses, diseases, disorders, or syndromes are the toughest task. There is no scientific process available to differentiate psychiatric illnesses. In the current scenario, no lab test exists in laboratory manuals. The documentation of neurobiological features, neurotransmitter pathways, structural abnormalities on neuroimaging, and genetic predispositions may be helpful in this concern 55. Such references may clarify prescriptions, treatment. Features of mechanisms underlying psychiatric comorbidities (mental illness, neuropsychiatric disorders, chronic inflammation, 56, alcohol abuse, personality disorders, anxiety, stress, neuropsychiatry disorders, eating disorders, neurodegeneration, and Axis II features), are the key components of any psychiatric diagnoses 57. Molecular imaging covers an unlimited span of research areas and touches the inside of the following fields i.e. molecular biology, bioengineering, nanotechnology, protein engineering, bioinformatics, genomics, transcriptomics, metabolomics, and proteomics 58. Thus, it can explore the ins and outs of therapeutics and theranostics (diagnose diseases, approach drug design, and assess therapies). Molecular imaging visualized the complicated biochemical transformation of the physiology and pathology of disease by targeting the molecular, biochemical reactions that represent the mechanism of the diseases. The expansion of nanotechnology is further shaping the edges of these imaging techniques 59. This is because of nanomaterials assembling with molecular imaging that further explores new frontiers with developmental abilities of the detection of biochemical compositions. Thus, the complexity of the new nanotechnologies is making things simpler to get better imaging and diagnosis 60. To diagnose the inflammation and neurodegeneration, the theranostics platforms must have to overcome the blood-brain barrier that is not workable because of the complex nature of these natural barriers, **Figure 5**. Thus, there is an urgent need to frame such strategies that must have a critical size and ability to remain stable during the cross over mechanisms. The innovation of nanotheranostics resolved this issue 61. These novel technical inventions are a wide range of nanoplatforms, nanocarriers, and nanodelivery agents i.e. nanocarriers, nanotube, nano-robots, miniatures, nanowires, nanospheres, nanoemulsions, and nanogels, 62-65, crystals, liposomes, micro-emulsions, solid lipid NPs, and hydrogels for an accurate diagnosis 66.

Implementing the novel concept of nanotechnology in neuroscience by using newly designed nanotheranostics and nanotherapeutics are the recent trends and new avenues for treating neurological and mental disorders. Some of them can act all together as nanotheranostics and nanotherapeutics, and such abilities very well appreciated and implemented in diagnosis and therapy 67. These strategies gained a lot of scientific interest and must implement for the diagnosis of neuropsychiatry. Authors are underlining the needs and proposing more scientific look on the unique properties of the nanocarrier as theranostic and therapeutic agents and their application in neuropsychiatry, neurological disorders, cognitive processing disorders, mental disorders and illnesses (dementia, brain injury, psychiatric manifestations, epilepsy, movements disorders, cerebrovascular accidents, degenerative disorders, and concerned domain of neuropsychiatry 68. A lot of efforts have made for the innovation of novel nanodiagnostics and nanotherapeutics to treat neuroglial alignment, but, still, there is a need to address the current challenges and to cover future perspectives for the innovation of novel nanotools and nanodevices for the diagnosis and treatment of the neurological ailments. Nanotheranostics used for molecular diagnosis, analysis, and measuring the biological routes in cells and tissues to explore pharmacogenomics, and pharmacoproteomic information and strategies with nanomolar sensitivity by considering environmental factors that responded towards therapy 69,70. Nanotheranostics offer advantages of high volume/surface ratio and multi-functionality by preventing unwanted side-effects. The imaging techniques (positron emission tomography 71-73, and single-photon emission computerized tomography, biomarkers labeled by γ-emitting radioisotopes), showed potential empowering the localization of the specific drug molecule at the targeted sites, cells, and tissues, and therefore at the nanoscale, have clinical applications in neuropsychiatric disorders 74. The hormone peptide traced is efficient at the nanoscale because of their higher binding affinities, easily direct neurotransmitter, and very helpful in the regulation and monitoring of various disorders (modulation of behavior, fierceness, stress, tension, anxiety, alcohol intake, and anorexia) 75.

Utilization of peptides in neuropharmaceuticals and nanotheranostics for diagnosis and cure of a wide variety of mental illness, disorders, and neuropsychiatry. Designed strategies of nanotheranostics based on peptides are recent developments for the innovations of tools that provide a lot of opportunities for enhancing further understanding to diagnose neuropsychiatric disorders. The tools of neuroimaging and electrophysiology detect the etiology and pathologies of CNS disorders and such efforts enhance the possibilities of exploring the mechanisms underlying inflammation and neurodegeneration 76. These novel modalities highly appreciated for intervening in newer theranostic conclusions. It is in demand to increase the understanding of the uncovered and untouched pathology of CNS disorders. The improved version of these technological findings for proper diagnosis and imaging are nanotheranostics tools. A combined effort of bioinformatics and up-to-date genomic methodologies will decisive in the innovation of these innovative modalities. Surface plasmon resonance approaches to include nanoparticles for the detection of Aβ and, to a lesser extent, of α-synuclein 77. Nanoplatforms induced gold and silver nanoparticles designed for biomedical applications, especially for the innovation of new strategies for molecular diagnosis with higher analytical capability, sensitivity with the possibility to use for neuropsychiatry 78. These nanodiagnostics tools possess very good optical and spectral properties i.e. intense localized surface plasmon resonance helps in surface modification. Such on-demand capabilities of diagnostics tools lead towards a comfortable approach to generate natural adaptabilities to work with biomolecules. These new platforms are proficient in definite molecular recognition. These efficient strategies to test molecular discovery restricted to nucleic acid (DNA, RNA) and protein-based assays. Recently, nano-based devices innovated for the diagnosis of mental illnesses 79. There are wearable electronic devices having electrodes for detecting electrical muscle signals or neuronal signals. These comprise metal and gel layers. With a top grade of biocompatibility, no resistance with great potential for very good detection.

## Nanotherapeutics for Neuropsychiatry: Healing insights

**Figure 6:**
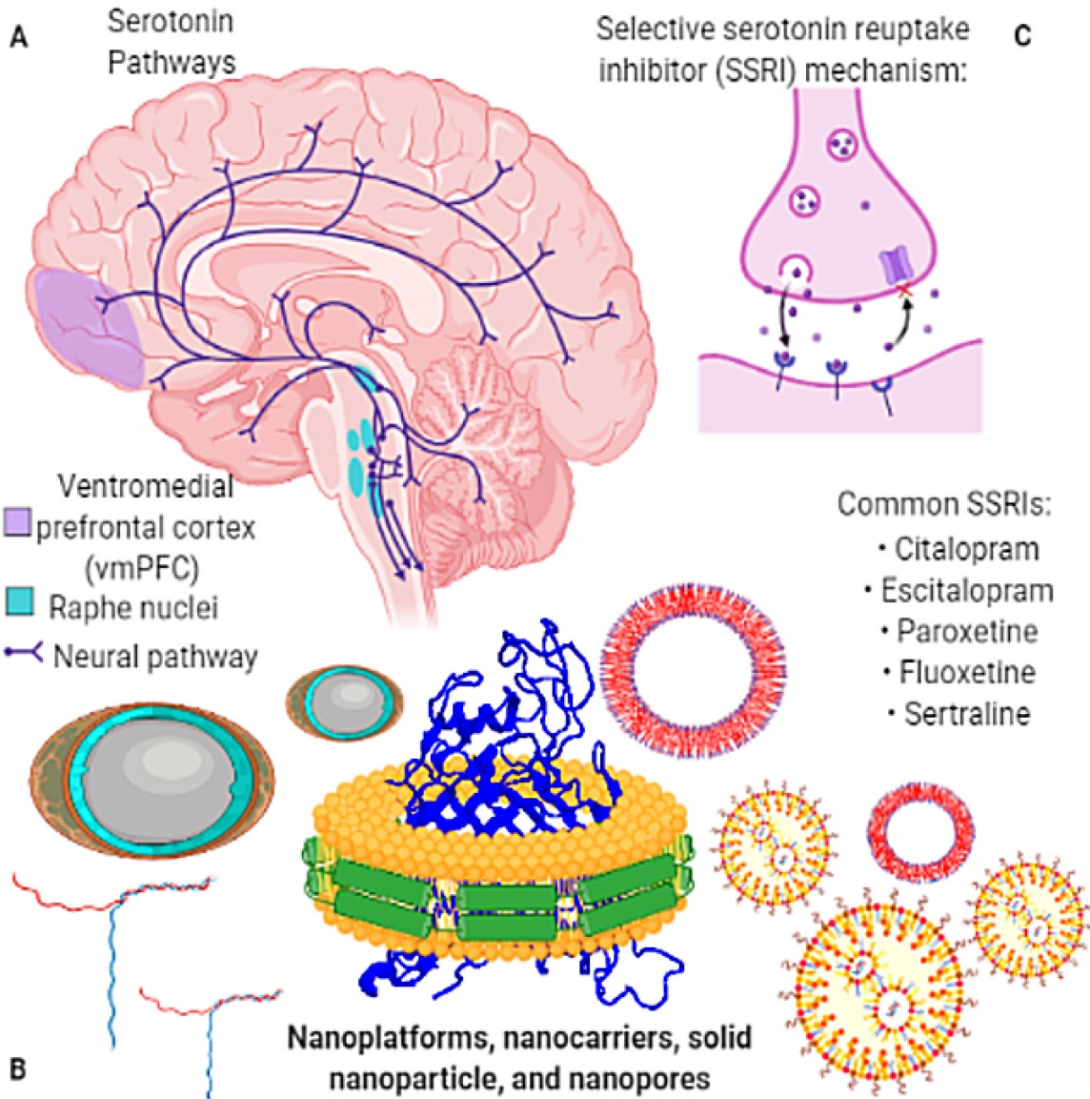
Nanotherapeutics for neuropsychiatry: Nanotherapeutics enabled with nanoemulsions, nanosuspensions, microemulsions, liposomes, nanobubbles, polymeric nanoparticle, or nanosomes dendrimers, solid-lipid nanoparticles, and polymeric micelles, showed promise for neuropsychiatric illnesses (depression, anxiety, and schizophrenia). The serotonin-related genetic variants to regulate stress-induced cortisol to activate the neural circuits direct mental illness. **(A)** Concerned pathways of the brain **(B)** Components of nanotools and nanodevices **(C)** Selective serotonin mechanism.

Neurological disorders, yet to diagnose fully, need original treatment strategies that remain in concern to heal mental illnesses. The major worries and successes always remain there in the conscious mind because sometimes drug deliveries fail, (unable to reach the site) and diagnosis (molecular imaging available) succeeds or vice versa because of existing multifaceted complications. Therefore, such an unclear phenomenon and an incomplete set of tools always push earlier diagnosis on the back foot. Therefore, the therapeutic option never comes out on both fronts 80. This is the chief cause of the failure of treatment. Again, it is acceptable that life-threatening coercions always remain lifelong. It is necessary to remind again about the major hurdle, the BBB because it is the chief cause for unsuccessful diagnosis and treatment. Thus, in the absence of a novel method, and the drugs used for the treatment methodologies underway unwanted neuroimmune activities, this is just happening because of unavailability of drugs or not fully formed nanoplatforms or integrated drug delivery used or unscientific strategies engaged in these multifaceted contests. It will use if these remedies for healing inside, then such steps could be fatal and may cause irreversible damages to neurons. The researchers have innovated recently nanoplatforms with a plan towards effective drug delivery strategies to treat CNS disorders creating no perturbation to the brain 81. Nanocarriers (liposomes, nanobubbles, polymeric nanoparticle, nanosomes, dendrimers, polymeric micelles, viral capsids) designed through computational modeling may do wonders by overcoming much-discussed huddle i.e. BBB 82. Drug degradation is also a major concern in these drug delivery systems. For targeting deeper sites, it must be a stable and highly designed drug molecule. So, these drug carriers must encapsulate. The delivery of nanocarriers to the CNS also hinder by the central nervous system (CNS) and BBB extracellular and intracellular barriers 83. To fulfill the need and the demand of such advanced delivery carriers with inbuilt abilities (high drug filling capability, targeted stroke, reduced nanotoxicity, and greater than before therapeutic effects), there is an urgent need to develop strategies and transform them into reality. Available preferred remedies are quite nearer to these proposed nanocarriers include different nanotherapeutics and nanotheranostics **Figure 6**. The upcoming challenges are there for the search for promising nanocarriers to heel neurological and psychiatric disorders, and their activities must be in the inner part of the CNS 84.

So, there is another concern that arose just because of alterations (in cellular processes, drug stability, cell organelles preferences), along with existing befell in pharmacokinetics and pharmacodynamics of the known therapeutics, mainly directly linked to the route of administration 85, and cellular transformations 86. Last, future perspectives and keenness for the discovering of nanotheranostics and nanotherapeutics as CNS/BBB drug delivery tools and devices will a major area of research in this field. There is a paucity of efficacious new compounds to treat neuropsychiatric disorders, such as antipsychotic drugs have some side effects (involuntary muscle movement, dystonia, metabolic disorders, and tardive dyskinesia). Receptor-mediated, transported medicated, or adoptive mediated transcytosis could be the mechanism through which a drug transported across the cell membrane by binding proteins or attached with the ligands for further pharmacological action 87. These ligands-drug binding formulations have implemented in treatment by transporting the drugs beyond BBB. The dominant ideology comes from the study of the drug target signaling network responses at the single-cell level and how the lymphocyte responds towards a drug. Therefore, the neuropsychiatric drug discovery method always considered such multifaceted mechanisms. During the procedure of such link establishment, the major happening here is primarily T-lymphocytes, which showed functional responses on all the sides of neuropsychiatric medications to establish targets by an emerging drug. These developed therapeutic targets come into existence during the phospholipase Cγ1-calcium signaling pathway to settle the progress of antipsychotic treatment of schizophrenia patients 88. Novel drugs like L-type calcium channel blockers and corticosteroids identified as the best therapy available and prefer to use during compound library screening. These scientific routes further exposed the consistent activities of neuronal cells as their responses. For speeding up the drug discoveries, there is an urgent need to enrich the forecast of the in-vivo effectiveness of the targeted drugs prescribed for neuropsychiatric condemnations 89.

Current therapeutics have a lack of qualities (great hepatic metabolism impact, and little half-lime) preferred as inbuilt properties to be present in perfect therapeutics prescribed for the neuropsychiatric illnesses (depression, anxiety, and schizophrenia). The efforts for the innovation of novel nanotherapeutics held up in the absence of perfect strategies. Nanomaterial induced nanocarriers and multifunctional nanoplatforms used in their designing might be successful and to consider as a key component in the innovation of nanoformulation with high treatment efficiencies 90. Such efforts will dilute all the challenges that existed in the middle of the path of discovery of these novel drug remedies for replacing psychotropic drugs. Nanotherapeutics enabled with nanoemulsions, nanosuspensions, microemulsions, liposomes, nanobubbles, polymeric nanoparticle, or nanosomes dendrimers, solid-lipid nanoparticles, and polymeric micelles showed tremendous promises and proof of this method evidenced in-vitro and in-vivo. Such advances are the key and the fostering steps in the cycle of discoveries required for potential clinical outcomes and benefits. These new drug therapies well equipped to treat neuropsychiatry and bipolar-like behaviors 91. To identify the gene responsible for and allied with these disorders, are a major concern, but these newly discovered tiny machines have the potential to recognize them with the ability to diagnose and identify the concentrations linked with these genes. The mechanisms happening in nanodomain existed between the area of communication and brain cells. These proteins sequencing signaling irregularities commonly define as synapses. The mechanism of a synapse regulated by gene formulation in the nanodomain constituents. Bipolar disorders and psychiatric illnesses cause uneven shifts in mood, activity, energy level, and day-to-day tasks 92. The stress and environmental factors directly influence the correct compositions of the genetic materials and degenerate it. However, there is an urgent need to specify psychiatric risk and gene degradation. Illumination and resolution techniques are the key sources for this kind of analysis. The domain area too discovered has a very fine architecture that falls to a very low range of nanoscale. The abnormalities of such bipolar disorders, neuropsychiatry illnesses, and leading risk genes interconnect phenomena. Therefore, the aim of nanotherapeutics to target these genes related issues and a novel procedure needed for healing insight are upcoming challenges.

## The monitoring of the drug release, *vivo* imaging, toxicity, current challenges and opportunities for future research strategies: Nanotherapy for Psychiatric Disorders

Nanomedicines, in nano-therapy, comprise nanomaterials, nano biosensors, and nanoscale biomaterials to perform as a vehicle and a tool to deliver and release the drugs at a precise specific target. The therapeutics to treat psychological disorders and mental illness 93 have not yet been successful because of the complications that existed in early diagnosis, frame long disease courses, and in addressing the challenges of drug delivery in crossing the biological barriers (blood-brain barrier) 84. Recently, nanotherapeutic emerged as a potential tool for precise diagnosis, efficient prescription, accurate imaging, and distinct psychiatric diseases. Hereabouts, this section comprises the advantages and disadvantages of nano therapy subsisted in psychiatric treatment and the underlying mechanisms of disease pathogenesis also outlined 94. It evidenced 95 that the accomplishment of nanodevices 95 and nanotools 96 in nanotherapy is an excellent approach for correct diagnosis and efficient therapy 97. Consequently, the nanomaterials possess an exceptional potential and proficiency for molecular/nano-scale interactions, therefore at the nanoscale, it must incorporate with theranostics and therapeutics for the diagnosis and repair of degraded neurons, cells, and tissues 98. Multifunctional nanomaterials included in nanomedicines magnify drug release, enhance the bioavailability, improve specific target delivery at the diseased locality, reliable surface functionalization, and capable to penetrate the BBB. Therefore, these nanotools and devices will at the forefront of diagnostic and therapeutic available as a possible best remedy for psychiatric diseases. Mental disorders are expanding day by day, but there is no further development in novel psychiatric medications. The discovery of nanotheranostics and nanotherapeutics is addressing the expectations and concerns outlined in the treatment of mental disorders 93. The paradigmatic crises addressed well by nanotechnology invented novel strategies to innovate specific therapeutic 99 to treat neurological 100 and psychiatric diseases 101. In the previous section, the authors conclude that nanotherapeutics are the best drugs and their carrier's healing psychiatry. Nanotherapeutic and nanotheranostic have infinite potential that is worthwhile and essential for enhancing therapeutic efficiency, and there is no toxicity. Subsequently, based on the sizes (1 and 100 nm) and shapes of the multifunctional nanomaterials, they consider the best resource to feature in drug delivery vehicles and prescribed as antipsychotics and antidepressant drugs 102. The variations of (size and shapes) exist in the nanotools and nanodevices, which execute and induce proficient features to cross the biological obstacles (blood-brain barrier). Remedies of nanotherapy derived from multifunctional nanoparticles perform as antipsychotic and anti-depressive agents to treat psychiatric diseases and disorders (schizophrenia, depression, and bipolar disorder) 103. Therefore, the nanotherapeutics and nanodiagnostics at the nanoscale have variation in design, and space in surface atoms and molecules, which transform them as the best theranostic and therapeutic.

The authors also critique the trends in psychiatric neuroprotection and address future trends and challenges by covering the aspects to theoretical frameworks based on psychiatric neuropathology and its applicability 104. The neuroprotective factors in psychiatry would be exceptionally pertinent to find novel treatments and expansion strategies. The nanoelectrodes or wearable sensors made of metal and outfitted with a gel layer capable of monitoring operations, identifying electrical muscle signals (electromyography), or neuronal signals (electroencephalography), and measure electrical conductivity by direct contact with the skin. These electrodes, wearable sensors, have biocompatibility, low communication permanence, and immense capability to adjust with the outline of the skin and hence able to detect the signal-to-noise-ratio 105. The detected biological signals used to identify the mental states, like neural, physiological, and muscle signals and portraiture of mental processes; therefore it has considerably enhanced psychic dysfunction analysis and operative repair. Shortly, nanotechnology will lead as a diagnostic (or neuroimaging) tool for assessing brain activity to identify psychiatric and neurological disorders. Further, nanotools will abolish depression and simultaneously intensify the intrinsic neurocognitive 106 potential by the atonement of multiple medications, boosting bioavailability, retention, and metabolization. Silica-based nanocarriers can deliver piracetam, a popular racetam nootropic, across the blood-brain barrier as a medicine. These nanodevices elaborate on the features of psychiatric drugs by enhancing their bioavailability to elicit an effect. The strategies of nanotherapy designed to treat psychiatric disorders also commit to boosting the absorption and as a result, it reduces side effects 107. For example, nanotechnology increases the efficacy of antidepressants, and then it offers a superior remedy that increases the potency of smaller doses. Therefore, the pharmaceutical enlarges nanopharmaceuticals engendering to treat mental illnesses. Nanotechnology ensures drugs optimally as it metabolizes and processed by the body. Such a drug delivery system illustrates minimal effective dose to the target area to minimize side effects. Nanorobots may contribute to generating scientific data concerning how does neurophysiology strengthens during therapy and help for more accurate diagnoses. Nanosensors test the efficacy of certain drugs and able to provide feedback on the drug's efficacy, potency, and effects 108. Nanosensors contribute in-depth concerning the antidepressant mechanism and psychotropic pathways offer entire patient-specific data. One of the original help benefits of nanosensors is that it allows for universal adoption quickly, safely, and effectively. It is possible to implant nanowires, a device only 200 nanometers in diameter, within the brain to read individualized signals of a patient suffering from depression. These nanowires, thin and flexible, will help to know how the researcher develops recent treatment and would not interrupt active neurons. These devices can collect data on neurotransmission 109 and nerve signals within the brain.

Nanobots will restore damaged tissue and neurons in the brain and are capable to excite definite sections of the brain artificially to neutralize depression and anxiety. Inserted probes or derived from nanomaterials to implant in neurons incite the happiest sites inside the brain to balance depressive signs. The intensifying brain illness could efficiently improve by the pre-programmed nanobots included into brains to restore openness, and employed in the development of artificial nano-replacements, promote stem-cells, renewal of faded cells and tissues 110. Unavoidably, there are noble concerns with the nano-neurological enrichment and will plead. Nano-scaled implants intentionally change the neural pathways in the brain to mimic receptor proteins and neural pathways. We reviewed it that certain implants proficient to intensify the release of neurotransmitters found in excitatory and inhibitory exercise. The initial implementation of nanotechnology in psychiatry assures to enhance the pharmacokinetic profile and effectiveness in various medications. Nanotools (liposomes, nanobubbles, nanoparticle polymers, or nanosomes) are capable to cross the blood-brain barrier with enhanced dose efficiency and dispense the drugs at specific neurons in the brain 111. The nanoshells and dendrimers have intensified potency, thus capable to gain more enhanced therapeutic effects. Specific rigidity and shapes of nanodevices and nanotools decide the interaction phenomenon between the drug molecules and cell membranes. Liposomes are highly capable of the ligand coupling. Nanosomes derived from supercritical fluid technology is the developed version of liposomes. The tiny carriers (nanotubes and polymeric nanoparticles) are efficient as a drug carrier to deliver drug molecules at a specific target within the brain by overcoming the blood-brain barrier and maximize drug discharge 112. It also identifies Nanobubbles as tiny bubbles capable of carrying gas molecules within a cavity immersed in a liquid solution to enhance the effectiveness of pharmaceutical drugs.

The tiny tools employed to seek space in the interworking of neural networks in the dense brain. Why is nanotechnology considered treating psychiatric illnesses 113, because, it (i) is highly capable to resolve the difficulties in neuropharmacology by overcoming the blood-brain barrier, (ii) can supply drugs at specifically targeted cells selectively and accurately (iii) has multiple classes of tiny tools (liposomes, nanosomes, nanoparticle polymers, nanobubbles) as drug delivery agent and always ready to perform various responsibility, (iv) can enhance drug bioavailability, and pharmacokinetics, (iv) can promote the effectiveness of psychotropic drugs with zero nanotoxicity, (v) especially, nanoshells and dendrimers can live analysis, and (vi) can yield specialized compensation *in vivo* imaging, metabolome analysis, and modeling of the central nervous system? It is clear from the reported literature that new nanoparticles assemblies able to create free-scale networking that will perform as artificial neural systems for the healing, repair, and functioning of synapses, diffusion of a neurotransmitter, and mimic synaptic behavior 114. Nanotools and nanodevices are proficient to create nanoparticle assemblies, recently termed as artificial intelligence, to treat mental illnesses and disorders. Every aspect of the nanotechnology look promising, but the safety issue is still not addressed fully. It intensified this as an ominous state of this technology. These innovative strategies used for the diagnosis and treatment of neuroinflammation, nerve injury, failure of neuronal networking, psychiatric conditions, and brain dysfunction 115. These achievements already show the perspicacity into the potential of this nanotechnology.

## Dysfunction of Neuronal Networks in brain function

What is the role of neuronal networks in brain functioning, how is it responsible for CNS disorders, and how is any therapeutics to apply if any dysfunction occurred? The most critical aspect is how the brain performs many of its major complicated tasks. We can only explain this via the investigation of neuronal network control 116 and network interactions 117. There is an enormous network that experiences important operative changes, resulting in substantially original brain functions. The abnormal interactions interrupt proper brain functioning, and the needed therapeutic to regularize dysfunction by network approach is the key feature of this review. Thus, the major aspects of neuroscience, pharmacology, and psychiatry (neurobiological disorders) covered to explore the control mechanisms, normal network function, strategies to improve therapies for brain disorders 118. The astrocytes, already illustrated in Figure 1, a glial cell, are useful portions of the synapses, reacting to neuronal activity and coordinating synaptic transmission and flexibility. Consequently, neuron-glia networks supervise processing, transference, storage, and communications of the information by the nervous system between astrocytes and neurons reside at the cellular and molecular levels but not yet fully discovered. It's evidence that astrocytes are integral components of nervous system networks, interlinked with intracellular signaling in neuron-glia networks 119, and to know it the patterns of structural connections in the brain which hold matchless feats of perception and a wide variety of behaviors 120. It is possible to do the mapping of these patterns by non-invasive imaging techniques. It is an urgent need to understand the interior of neurons structure, neuron networking, physiology, and functioning style and how it care cognitive processes, such efforts will crucial for the designing and development of simulation-based therapies for mental illness and psychiatric disease.

The communication between complicated architecture (neurons and brain regions) concerns energy minimization, and information transfer 121. The materialization of wide-range exchanges and synchronization from the combined firing of specific neurons implore impressions of emergence and criticality from mathematical mechanics. By using the theories of network control, the designing strategies for the innovation of nanotheranostic to cure cognitive disorders can accomplish in nanotherapy. Therefore, the need to highlight and outline the effectiveness of nanotechnology-enabled procedures and techniques 122 (electrophysiology and intracellular sampling) to understand the brain and its components are there 123. Implementing nanotheranostics techniques to interrupt the brain, explore breakthroughs to get single-cell resolution, for better diagnosis, and monitoring of neurological diseases. Nanotools 124 have the potential to transport therapeutic and imaging contrast agents into the neurons and nerve cells by overcoming the blood-brain barrier, clear from the cited literature 125. Nanotools and nanodevices can increase drug-drug interactions, facilitate pathogen clearance, and promote the transportation of biologically active molecules to achieve better signal transduction in the immune system. The key to the success of nanotechnology linked with the many aspects and surface modifications^126^,^127^ is one of them, which can engineer these multifunctional nanoparticles to enhance targeted cellular uptake, and enhance the pharmacokinetic properties (distribution, and excretion) 128. Molecular imaging (nanotheranostics) used 129 to determine the biochemical and pharmacological routes at the nanoscale *in-vivo* for most of the neuropsychiatric disorders 130.

## The mind knockouts to kill the main Devil: Strategies for killing the stress

In modern-day nanomedicine, there is an enormous need for indispensable tools in disease monitoring and therapy at the nanoscale, and the “engineered” nanomaterials have tuned to a harmonious set of tools as required. Thus, this modality has progressed to gain significant attention in the neurotherapeutics sciences concerning to their versatility of tunes characteristics 131. Briefly, it can determine the advancement in the field via an emphasis on core properties on these engineered nanoplatforms. Further, modulation of surface modification and functionalization has assisted novel drug delivery of the therapeutics remedies. In developing these drug delivery modalities, challenges such as the testing of definite drug delivery, monitoring drug release, and inhibiting opsonization emerge as significant factors meant to affect the outcomes 132. Therefore, it turns out to be more prominently essential to understand the different molecular mechanisms alongside the development of carrier vehicles. These are essential factors for how nanocarriers hold the drug-load, drug-bioavailability, and mechanism of drug targeting. Further, other important aspects of nanoplatforms administration-routes need to address as these have to face natural hurdles, and those are the key challenges that must be an emphasis on drug transportation beyond biological and other barriers 133. The chief objectives of neuro-delivery systems must look into the key aspects of it, i.e. to explore the neurogenomics, diagnosis, discovery of potential therapeutics, and disorders of the nervous system. Thus, a better straightforward insight of nanoplatforms architecture and trials needed for the discovery of better drug delivery vehicles that address the key issues. These complications will be further led by the explorations using next-generation sequencing, genomic biomarkers, brain mapping, and molecular diagnostics to find out the efficacy of novel nanotherapeutics for healing neurologic disorders. Implementation of the Knowledge of nanotheranostics and nanotherapeutics will improve disease diagnosis, imaging of neurodegenerative medicine, and treatment interface while handling and dealing with neuropsychiatry and mental illnesses 134.

It represents stress as a sequence of incidences of physical, chemical, and emotional phenomena involving the brain. Mental tension generated this. Stress could be a revolving factor to generate complex diseases. The sequencing of these incidents produces a lot of biological and chemical entities that promote various factors to imbalance the body's equilibrium. Emotional factors and originated harmful chemicals affect the nervous system. Overall, it is tough to distinguish stress and fear, and the same huddle to pinpoint the diseases. A three-dimensional approach needed here for healing the wounds of memory. On one side, there is a need for an exact differentiation of the symptoms to define the psychological imbalance, and the on the other side, there is a requirement of the evaluation requires for a proper nanotheranostics technique to diagnose the nanodomain, that exist in the stress's territory devil or where are the damaged neurons 32. In the third dimension, there is a search for the best remedy for the ultimate attack on the main nanodomain by nanotherapeutics for the repair and regeneration of the damaged neurons. Overall, three dimensional therapeutic and diagnostic efforts will kill all causes of neurodegenerative, neuropsychiatric, and bipolar disorders. The aim must be to harness the qualities of the nanomaterials for better diagnosis, to explore cellular microenvironment for better identification of the nanodomain of the diseases, and this lead to finally the most and formalized efforts to enhance the efficiency of drug delivery to the brain for better regulation to the cellular microenvironment. A lot of variety of nanomaterials exist with unique abilities and capabilities which may use for molecular imaging, diagnosis, and treatment of neurodegeneration disorders. The best-identified nanoplatforms will be ultimately used in the designing of novel therapeutics to provide better clinical therapies. The shortcomings will further need to eradicate in the path towards avoidance of disturbance in our vision of final applicable nanotheranostics for neurodegenerative diseases 135. Therefore, the physiology of the diseases and the weakness of the nanoplatforms (lipid-based, polymer, metal, silica, and hydrogel nanoparticles) must re-evaluate 136. The up-gradation of the current strategies will be fruitful for a successful therapeutic remedy. Now, the primary emphasis must be on how these must co-relate to psychology, neuropsychiatry, nanotechnology, theranostics, and therapeutics to improve clinical outcomes.

Glial cells are the key component in the brain for responding to the brain's response 137. Other cell organelles also have their monitor forefronts for their contribution to the cell functioning. The biochemistry of the cellular details must be there with all the aspects, while it uses such strategies. The features of cell components must separately outline in every aspect. The understanding of complete nanodomain territories facilitates the designing of systems towards the controlling impact on the end target leading further to generate and govern cellular responses. Scale down nanofluidic tools and assemblies that transport fluids proficiently to the location stimulated or damaged cells or tissues or targeted sites have potential in regulating chemical and physiological change at the site of end affected tissue partially. More efficient site-specific targeting by the use of targeting moieties as an addition to nanodrugs has well studied, resulting in reduced systemic side effects and improve patient compliance. Closed-looped drug carriage nanodevices and tissue transplants comprising sensors on the similar chips are the recently advanced nanosystems that have shown success in obtaining the desired end impact towards mitigation of respective malignancy. Further, the nano/micro-surgical devices, nanorobots, nano-molecular motors, or nanobots proficient for navigating all over the body for healing damaged cells and tissues are the emerging most efficient nanodevices or nanosystems that can be used to destroy tumors or viruses and even can help in performing the regulated gene therapy. In neurotherapeutics development, the Nanorobots, in particular, will have unprecedented potential in drug delivery to brain cells aiming for precise local targets. Application, the Nanobots made as 'DNA-bots': i.e. nanorobots made of DNA, that release the tethered drug (small interfering RNA (siRNA), DNA fragment, and/or another chemical drug) to the environment when triggered by electromagnetic radiation 138. Promising future medical technology that can gain, transmit, and save single-neuron electrical evidence resultant of a synaptic deal with spike frequency can provide with an ultimate tool called 'Neuronanorobots'. These nanorobots map various information channels in real-time *in vivo* in the human brain. Mechanically actuated DNA origami robots are potential thought-controlled nanobots that can alter the response to a brain's cognitive state. This benefits the therapeutic control in disorders such as schizophrenia, depression, and attention deficits, which are among the most challenging conditions to diagnose and treat 139.

## Lack of awareness into Psychiatric Illness: A critical review

The symptoms of psychiatric illnesses have similarities with a terrible reputation of unwarranted behavior or cannot say it is just dangerous behavior. If such symptoms like hallucinations, confusion, suicidal thoughts, delusions, depressed mood, rapidly fluctuating mood, memory changes, incoherent thought patterns, elevated mood, addictive behaviors, are there then there is a need for accurate analysis of the symptoms and allied specific patterns of symptoms. Patients with psychiatric illnesses often have impaired insight, which means they have a diminished ability to understand the nature of the disorders. To find out a pattern of mental illnesses and the nature of a particular illness are two unique kinds of situations. Such complications make it even more complicated 140. The affected person initially confused that he is sick or is normal. The interpretation is, the patients “may not understand the mechanisms underlying their illnesses, and this causes the treatment more challenging, or during these psychiatric illnesses, the patients rarely recognize that anything is wrong 141. So a lack of insights, creating such troubles that define as anosognosia, the initial phase of severe mental illness which impair the capability of the concerned person to know and recognize someone's illness 142. A person suffering from schizophrenia may not understand that the voices and delusions are not real, or in the same case of a person having severe depression may not understand why others don't understand him. More complicated examples are from the mental illness of severe dementia. In this disorder, patients think they can completely do everything. Proper functioning of the brain based on a complex set of brain calculations and any defects or failure or dysregulation of these mechanisms caused psychiatric illnesses 143. It involves a pathological mechanism in it, and this condition interferes with motivational, cognitive, and emotional brain systems lead to dysregulation of the brain systems underlying insight regulation. Therefore, the affected person has to face a state of high internal emotion and low motivation. This breaks the equilibrium of normal functioning. To avoid anosognosia or similar conditions, there is a need for a proper insight (higher-order brain networks underlying attention, cognitive control, and working memory). The concept of cognitive is helpful, which encourages the affected person to improve his attention, ability, trust, a memory that is helpful to improve the insight 144. Thus, anosognosia may cause more complications in psychiatric illnesses because an inaccurate insight has misperceptions, and this starts conflicts, and finally, anxiety reached the top level. In such situations, the patients avoid treatment or to stop taking their medications. Variations in awareness tactics will dilute the complications of anosognosia and accordingly patients respond to it accepting the persistence of mental illnesses (psychosis, depression, personality disorder, obsessive-compulsive disorder, paraphilic disorders, and substance addictions). With Schizophrenia, matured awareness will push not to accept the diseases towards the backside by exploring the possibilities depend on the verities of options for solution and which one to adopt i.e. neuroscience, psychiatry, psychology, social-cultural/anthropological studies, linguistics and philosophy 145. The etiology helped to find out the reasons for neuropsychiatry disorder, Schizophrenia, and to search the strategies for treatment linked directly with the processes i.e. reformulation, understanding of causation, and treatment method 146. Therefore, the dimensions of evaluation with an outcome provide a platform to bring actual issues into the light, and many aspects of lack of insight is a line of attack. Insight, denial, the mental representation of the self, and psychiatric disorders are interrelations phenomenon, wherein every aspect linked with each other closely, and such interpretations will diagnose immediately, a person with psychosis.

## Conclusion and Outlook

Because of the inflammation, undesirable damages start at the cellular defense system, which was duly opposed by initiating an immune response to protect from pathogenic infection, to prevent tissue damage, to hinder autoimmune signals within neurons. The fight starts with an imbalance in neuron cells caused by diseases. With uncontrolled inflammation as the prolonged one, it starts chronic diseases (autoimmune diseases, mental illness, ischemic disease, and systemic inflammatory response syndrome). And there are also a lot of diverse phenotypic consequences as dysregulation of immune homeostasis 147. The efficacious diagnostic and efficient therapeutic agents, as (immunotherapies and gene therapy), can only investigate these conditions to target the exciting insight by the protein delivery. Further, it will amaze to analyze how they modulate the immune homeostasis to achieve remission of chronic inflammation. The immunomodulation therapies used through engineered autologous to the immune cells for correcting the disease phenotypes to achieve the needed outcome. These are the only scientifically proved remedy available. These therapeutics having tissue-targeting precision presented efficient therapeutic strategic choices base on cell membrane-nanotherapeutics correlation. This remedy looks scientifically more appropriate because the interactions between their surface components and those of the target cells can achieve the targeting capability. In this way of treatment, the best part of it, eligible for the delivery payload at the inflamed site. It is very well to accept that nanoformulation has potential in modulating inflammation. Nanotherapeutics induced with the cell membranes of cells involved in the inflammatory progression more efficiently used for effective targeted transport to inflamed tissues 148. The innovative technology is "nanotheranostics, “which crosses all the hurdles, not properly addressed in the development of nanomedicines and albeit with many limitations to overcome 149,150. A new variety of nanotheranostics of silica-coated bismuth-ferrite, are light-responsive caged molecular cargos, be easily activated with near-infrared light, and imaged at longer wavelengths for both detection and drug release processes 151. Once light-triggered, these newly innovated nanotherapeutic agents release the drug, monitored, and quantified the release by recording the release inbuilt in these platforms as imaging-diagnosis. This method fulfills the need for the on-demand release of the drug, which was the much more awaited requirement. The other issue addressed during the innovation of this technology is that they are highly capable to save the decoupled imaging in tissue depth. It must use these nano smart devices in axonal regeneration, neurological operations, and molecular imaging and CNS imaging 152. These nanoplatforms may cause a lit bit of oxidative stress, autophagy, and lysosome dysfunction, and signaling pathways blocking, so they have very low nanoneurotoxicity.

Side effects of drugs, hundreds of metals, particulate, and volatile organic compounds, environmental pollutants, metal particles, and chemicals, can cause cardiovascular diseases, mental illnesses, brain hemorrhage, neurodegeneration, and dysfunction of CNS mechanisms. Ultra-fine particles triggered oxidative stress and neuroinflammation, which further affect the white matter of the neurons in the brain 153. The cognitive deficits and behavioral impairment effect of brain-neuron connectivity. Finally, impairments of the parietal and temporal lobe functions of the brain neurons lost, by the impact of the mechanisms (protein aggregation, microglial activation, apoptosis oxidative stress injury, neuroinflammation, and mitochondrial dysfunction), triggered by neurodegeneration and as a result neuron disabled their routine functioning 154. The therapeutic aim is to attain a significant drug distribution and transportation in the brain cells and tissues to gain desired therapeutic outcomes. Capabilities of the nanoplatforms i.e. minute size, tailored with functional modalities, and drug release at specific sites declared them as the best nanotherapeutics and nanotheranostics agents that can target the brain and CNS. These remedies further categorized into polymeric, lipids, and amphiphilic nanocarriers and may inject through various routes to prove their ability as best targeted therapy agents 155. These approvals are necessary to understand their highly implemented mechanism and their successful delivery route. This kind of analysis is a must because the defensive barriers obstruct the treatment remedy timely. This is understandable and very useful to treat mental illnesses (depression, anxiety disorders, addictions, personality disorders, schizophrenia, eating disorders, dementia, and panic disorders), and meningitis. Such diseases can only be treated if the drug delivery is target specific to achieve a required therapeutic outcome.

## References

[1] Voet S, Srinivasan S, Lamkanfi M, Loo G (2019). Inflammasomes in neuroinflammatory and neurodegenerative diseases. EMBO Mol Med.

[2] Lassus B, Magnifico S, Pignon S, Belenguer P, Miquel M-C, Peyrin J-M (2016). Alterations of mitochondrial dynamics allow retrograde propagation of locally initiated axonal insults. Sci Rep.

[3] Cervenka I, Agudelo LZ, Ruas JL (2017). Kynurenines: Tryptophan's metabolites in exercise, inflammation, and mental health. Science.

[4] Rosell DR, Futterman SE, McMaster A, Siever LJ (2014). Schizotypal Personality disorder: a current review. Curr Psychiatry Rep.

[5] Arns M, Drinkenburg WH, Fitzgerald PB, Kenemans JL (2012). Neurophysiological predictors of non-response to rTMS in depression. Brain Stimul.

[6] Gonda X, Pompili M, Serafini G, Carvalho AF, Rihmer Z, Dome P (2015). The role of cognitive dysfunction in the symptoms and remission from depression. Ann Gen Psychiatry.

[7] Picard M, McEwen BS (2014). Mitochondria impact brain function and cognition. Proc Natl Acad Sci.

[8] Benbrika S, Desgranges B, Eustache F, Viader F (2019). Cognitive, Emotional and psychological manifestations in amyotrophic lateral sclerosis at baseline and overtime: a review. Front Neurosci.

[9] Pogoda K, Janmey PA (2018). Glial Tissue mechanics and mechanosensing by Glial Cells. Front Cell Neurosci.

[10] Ishii H, Yoshida M (2010). Inflammatory cytokines. Nippon rinsho Japanese J Clin Med.

[11] Brigida A, Schultz S, Cascone M, Antonucci N, Siniscalco D (2017). Endocannabinod signal dysregulation in autism spectrum disorders: a correlation link between inflammatory state and neuro-immune alterations. Int J Mol Sci.

[12] Kumar N, Kumar R (2014). Nanotechnology and nanomaterials in the treatment of life-threatening diseases. Elsevier.

[13] Dimitrijevic I, Pantic I (2014). Application of nanoparticles in psychophysiology and psychiatry research. Rev Adv Mater Sci.

[14] Chhikara BS, Varma RS (2019). Nanochemistry and nanocatalysis science: research advances and future perspectives. J Mater Nanosci.

[15] Kant V (2017). Revisiting the technologies of the old: a case study of cognitive work analysis and nanomaterials. Cogn Technol Work.

[16] Sullivan GP, Davidovich PB, Sura-Trueba S, Belotcerkovskaya E, Henry CM (2018). Identification of small-molecule elastase inhibitors as antagonists of IL-36 cytokine activation. FEBS Open Bio.

[17] Amor S, Puentes F, Baker D, Van Der Valk P (2010). Inflammation in neurodegenerative diseases. Immunology.

[18] Liddelow SA, Guttenplan KA, Clarke LE, Bennett FC (2017). Neurotoxic reactive astrocytes are induced by activated microglia. Nature.

[19] Lin MT, Beal MF (2006). Mitochondrial dysfunction and oxidative stress in neurodegenerative diseases. Nature.

[20] Niedzielska E, Smaga I, Gawlik M, Moniczewski A, Stankowicz P (2016). Oxidative stress in neurodegenerative diseases. Mol Neurobiol.

[21] Hamm G, Pamelard F, Bonnel D, Legouffe R, Hochart G, Stauber J (2015). Poster Abstracts. Drug Metab Rev.

[22] Mishra D, Fatima A, Singh R, Munjal NS, Mehta V, Malairaman U (2019). Design, synthesis and evaluation of Coumarin-Phenylthiazole conjugates as cholinesterase inhibitors. Chem Biol Lett.

[23] Singh R, Geetanjali G, Sharma N (2014). Monoamine Oxidase Inhibitors for Neurological Disorders: A review. Chem Biol Lett.

[24] Fiskum G, Starkov A, Polster BM, Chinopoulos C (2003). Mitochondrial mechanisms of neural cell death and neuroprotective interventions in Parkinson's disease. Ann N Y Acad Sci.

[25] Yakes FM, Van Houten B (1997). Mitochondrial DNA damage is more extensive and persists longer than nuclear DNA damage in human cells following oxidative stress. Proc Natl Acad Sci U S A.

[26] Zimmermann KC, Green DR (2001). How cells die: Apoptosis pathways. J Allergy Clin Immunol.

[27] Chhikara BS, Rathi B, Parang K (2019). Critical evaluation of pharmaceutical rational design of Nano-Delivery systems for Doxorubicin in Cancer therapy. J Mater Nanosci.

[28] Jin W, Qazi TJ, Quan Z, Li N, Qing H (2019). Dysregulation of Transcription Factors: A Key Culprit Behind Neurodegenerative Disorders. Neuroscientist.

[29] Behl B, Papageorgiou I, Brown C, Hall R, Tipper JL (2013). Biological effects of cobalt-chromium nanoparticles and ions on dural fibroblasts and dural epithelial cells. Biomaterials.

[30] Hammond TR, Marsh SE, Stevens B (2019). Immune Signaling in Neurodegeneration. Immunity.

[31] Bajwa E, Pointer CB, Klegeris A (2019). The Role of mitochondrial damage-associated molecular patterns in chronic neuroinflammation. Mediators Inflamm.

[32] Kovacs G (2016). Molecular Pathological classification of neurodegenerative diseases: turning towards precision medicine. Int J Mol Sci.

[33] Culmsee C, Michels S, Scheu S, Arolt V, Dannlowski U, Alferink J (2019). Mitochondria, microglia, and the immune system - how are they linked in affective disorders?. Front Psychiatry.

[34] Voet S, Srinivasan S, Lamkanfi M, Loo G (2019). Inflammasomes in neuroinflammatory and neurodegenerative diseases. EMBO Mol Med.

[35] Benedetti E, Cristiano L, Antonosante A, d'Angelo M, D'Angelo B (2017). PPARs in neurodegenerative and neuroinflammatory pathways. Curr Alzheimer Res.

[36] Liu C-Y, Wang X, Liu C, Zhang H-L (2019). Pharmacological targeting of microglial activation: new therapeutic approach. Front Cell Neurosci.

[37] Liu J, Chen Y, Wang G, Lv Q, Yang Y, Wang J (2018). Ultrasound molecular imaging of acute cardiac transplantation rejection using nanobubbles targeted to T lymphocytes. Biomaterials.

[38] He C, Klionsky DJ (2009). Regulation mechanisms and signaling pathways of autophagy. Annu Rev Genet.

[39] Cheung W, Kotzamanis G, Abdulrazzak H, Goussard S, Kaname T (2012). Bacterial delivery of large intact genomic-DNA containing BACs into mammalian cells. Bioeng Bugs.

[40] Rigby RE, Rehwinkel J (2015). RNA degradation in antiviral immunity and autoimmunity. Trends Immunol.

[41] Graeber MB, Li W, Rodriguez ML (2011). Role of microglia in CNS inflammation. FEBS Lett.

[42] Wong AD, Ye M, Levy AF, Rothstein JD, Bergles DE, Searson PC (2013). The blood-brain barrier: An engineering perspective. Front Neuroeng.

[43] van Horssen J, van Schaik P, Witte M (2019). Inflammation and mitochondrial dysfunction: A vicious circle in neurodegenerative disorders?. Neurosci Lett.

[44] Gerasimenko J V, Gerasimenko O V, Palejwala A, Tepikin A V, Petersen OH, Watson AJM (2002). Menadione-induced apoptosis: Roles of cytosolic Ca2+ elevations and the mitochondrial permeability transition pore. J Cell Sci.

[45] Chivet M, Javalet C, Laulagnier K, Blot B, Hemming FJ, Sadoul R (2014). Exosomes secreted by cortical neurons upon glutamatergic synapse activation specifically interact with neurons. J Extracell Vesicles.

[46] Ding HM, Ma YQ (2018). Computational approaches to cell-nanomaterial interactions: Keeping balance between therapeutic efficiency and cytotoxicity. Nanoscale Horizons.

[47] Arora G, Damle NA (2018). Radiopharmaceuticals for diagnosis of Primary Hyperparathyroidism. Chem Biol Lett.

[48] Bernardi P (1999). Mitochondrial transport of cations: Channels, exchangers, and permeability transition. Physiol Rev.

[49] Kiernan EA, Smith SMC, Mitchell GS, Watters JJ (2016). Mechanisms of microglial activation in models of inflammation and hypoxia: Implications for chronic intermittent hypoxia. J Physiol.

[50] Theam OC, Dutta S, Sengupta P (2020). Role of leucocytes in reproductive tract infections and male infertility. Chem Biol Lett.

[51] Irez T, Bicer S, Sahin E, Dutta S, Sengupta P (2020). Cytokines and adipokines in the regulation of spermatogenesis and semen quality. Chem Biol Lett.

[52] Huis In &apos;T Veld R, Storm G, Hennink WE, Kiessling F, Lammers T (2011). Macromolecular nanotheranostics for multimodal anticancer therapy. Nanoscale.

[53] Kroemer G, Galluzzi L, Brenner C (2007). Mitochondrial membrane permeabilization in cell death. Physiol Rev.

[54] Kumar N, Kumar R (2014). Nanomedicine for Neurological Disorder. In: Nanotechnology and nanomaterials in the treatment of life-threatening diseases. Elsevier.

[55] Sulzer D, Mosharov E, Talloczy Z, Zucca FA, Simon JD, Zecca L (2008). Neuronal pigmented autophagic vacuoles: Lipofuscin, neuromelanin, and ceroid as macroautophagic responses during aging and disease. J Neurochem.

[56] Dabur R, Sharma B, Mittal A (2018). Mechanistic approach of anti-diabetic compounds identified from natural sources. Chem Biol Lett.

[57] Minen MT, De Dhaem OB, Van Diest AK, Powers S, Schwedt TJ (2016). Migraine and its psychiatric comorbidities. J Neurol Neurosurg Psychiatry.

[58] Li S, Todor A, Luo R (2016). Blood transcriptomics and metabolomics for personalized medicine. Comput Struct Biotechnol J.

[59] Zarghi A, Zali A, Ashrafi F, Moazzezi S (2015). Abstracts of the 2nd International Conference on Behavioral Addictions - March 16-18, 2015, Budapest, Hungary. J Behav Addict.

[60] Wolf SA, Boddeke HWGM, Kettenmann H (2017). Microglia in physiology and disease. Annu Rev Physiol.

[61] Janjic JM, Gorantla VS (2017). Peripheral nerve nanoimaging: monitoring treatment and regeneration. AAPS J.

[62] Roy I, Anuradha A (2016). Synthesis and characterization of iron phosphate NPs and applications in magnetically guided drug delivery. J Mater Nanosci.

[63] Mittal P, Singh S, Singh A, Singh IK (2020). Current advances in drug delivery systems for treatment of Triple negative breast cancer (TNBC). Chem Biol Lett.

[64] Pant P, Gupta C, Kumar S, Grewal A, Garg S, Rai A (2020). Curcumin loaded Silica Nanoparticles and their therapeutic applications: A review. J Mater Nanosci.

[65] Kumari P, Gautam R, Milhotra A (2016). Application of Porphyrin nanomaterials in Photodynamic therapy. Chem Biol Lett.

[66] Zagrean AM, Hermann DM, Opris I, Zagrean L, Popa-Wagner A (2018). Multicellular crosstalk between exosomes and the neurovascular unit after cerebral ischemia. therapeutic implications. Front Neurosci.

[67] Xiao YF, Chen JX, Li S, Tao WW, Tian S (2020). Manipulating exciton dynamics of thermally activated delayed fluorescence materials for tuning two-photon nanotheranostics. Chem Sci.

[68] Cole JH, Marioni RE, Harris SE, Deary IJ (2019). Brain age and other bodily 'ages': implications for neuropsychiatry. Mol Psychiatry.

[69] Alharbi KK, Al-sheikh YA (2014). Role and implications of nanodiagnostics in the changing trends of clinical diagnosis. Saudi J Biol Sci.

[70] Kumar R, Sharma M (2018). Herbal nanomedicine interactions to enhance pharmacokinetics, pharmaco-dynamics, and therapeutic index for better bioavailability and biocompatibility of herbal formulations. J Mater Nanosci.

[71] Jain A, Kameswaran M, Pandey U, Sharma R, Sharma HD, Das A (2018). Synthesis and evaluation of a novel 68Ga-NODAGA-Erlotinib analogue towards PET imaging of Epidermal Growth Factor Receptor over-expressing cancers. Chem Biol Lett.

[72] Chen Q, Wang P, Low PS, Kularatne SA (2014). Recent advances in PET imaging of folate receptor positive diseases. Chem Biol Lett.

[73] Mishra AK (2018). Nuclear Medicine advances in development of radiopharmaceuticals for Scintigraphy, Positron Emission Tomography and Radiotherapy. Chem Biol Lett.

[74] Gorin JB, Ménager J, Gouard S, Maurel C, Guilloux Y (2014). Antitumor immunity induced after α irradiation. Neoplasia (United States).

[75] Bernstein HG, Dobrowolny H, Bogerts B, Keilhoff G, Steiner J (2019). The hypothalamus and neuropsychiatric disorders: psychiatry meets microscopy. Cell Tissue Res.

[76] Glass CK, Saijo K, Winner B, Marchetto MC, Gage FH (2010). Mechanisms underlying inflammation in neurodegeneration. Cell.

[77] Xia Y, Padmanabhan P, Sarangapani S, Gulyás B, Vadakke Matham M (2019). Bifunctional Fluorescent/Raman nanoprobe for the early detection of amyloid. Sci Rep.

[78] Yoo YK, Kim G, Park D, Kim J, Kim Y (2020). Gold nanoparticles assisted sensitivity improvement of interdigitated microelectrodes biosensor for amyloid-β detection in plasma sample. Sensors Actuators B Chem.

[79] Vio V, Marchant MJ, Araya E, Kogan MK (2017). Metal Nanoparticles for the treatment and diagnosis of neurodegenerative brain diseases. Curr Pharm Des.

[80] Sepede G, Sarchione F, Matarazzo I, Di Giannantonio M, Salerno RM (2016). Premenstrual dysphoric disorder without comorbid psychiatric conditions: A systematic review of therapeutic options. Clin Neuropharmacol.

[81] Heiss CN, Olofsson LE (2019). The role of the gut microbiota in development, function and disorders of the central nervous system and the enteric nervous system. J Neuroendocrinol.

[82] Anticevic A, Murray JD (2017). Computational psychiatry: Mathematical modeling of mental illness. Computational Psychiatry: Mathematical Modeling of Mental Illness.

[83] Saeedi M, Eslamifar M, Khezri K, Dizaj SM (2019). Applications of nanotechnology in drug delivery to the central nervous system. Biomed Pharmacother.

[84] Umlauf BJ, Shusta E V (2019). Exploiting BBB disruption for the delivery of nanocarriers to the diseased CNS. Curr Opin Biotechnol.

[85] Dahiya S, Kaushik A, Pathak K (2019). Formulation optimization of multicomponent aqueous coground mixtures of Meloxicam for dissolution enhancement. Chem Biol Lett.

[86] De Vries H, Van der Poll HM (2018). Cellular and organisational team formations for effective Lean transformations. Prod Manuf Res.

[87] Hervé F, Ghinea N, Scherrmann JM (2008). CNS delivery via adsorptive transcytosis. AAPS J.

[88] IRCT20120314009297N5. The effect of nano curcumin in male patients with residual schizophrenia.

[89] Giuliano S, Agresta AM, De Palma A, Viglio S, Mauri P (2014). Proteomic analysis of lymphoblastoid cells from nasu-hakola patients: a step forward in our understanding of this neurodegenerative disorder. PLoS One.

[90] Lu X, Liu J, Wu X, Ding B (2019). Multifunctional DNA origami nanoplatforms for drug delivery. Chem - An Asian J.

[91] M (2017). Metcalfe S, Bickerton S, Fahmy T. Neurodegenerative disease: a perspective on cell-based therapy in the new era of cell-free nano-therapy. Curr Pharm Des.

[92] Geriani D, Savithry KSB, Shivakumar S, Kanchan T (2015). Burden of care on caregivers of schizophrenia patients: A correlation to personality and coping. J Clin Diagnostic Res.

[93] Ei Thu H, Hussain Z, Shuid AN (2018). New insight in improving therapeutic efficacy of antipsychotic agents: an overview of improved *in vitro* and *in vivo* performance, efficacy upgradation and future prospects. Curr Drug Targets.

[94] Olloquequi J, Cornejo-Córdova E, Verdaguer E, Soriano FX, Binvignat O, Auladell C (2018). Excitotoxicity in the pathogenesis of neurological and psychiatric disorders: Therapeutic implications. J Psychopharmacol.

[95] Fond G, Macgregor A, Miot S (2013). Nanopsychiatry-The potential role of nanotechnologies in the future of psychiatry: A systematic review. Eur Neuropsychopharmacol.

[96] Margret A (2016). Stratagems of nanotechnology augmenting the bioavailability and therapeutic efficacy of traditional medicine to formulate smart herbal drugs combating. In: Pharmaceutical Sciences: Breakthroughs in Research and Practice.

[97] Hosseini Y, Alavi SE, Akbarzadeh A, Heidarinasab A (2016). Improving lithium carbonate therapeutics by pegylated liposomal technology: an *in vivo* study. Comp Clin Path.

[98] Uchiyama Y, Koike M, Shibata M, Sasaki M (2009). Chapter 3 Autophagic Neuron Death. Methods Enzymol.

[99] Pellegrini C, Fornai M, Antonioli L, Blandizzi C, Calderone V (2019). Phytochemicals as novel therapeutic strategies for NLRP3 inflammasome-related neurological, metabolic, and inflammatory diseases. Int J Mol Sci.

[100] Choudhury A, Sahu T, Ramanujam PL, Banerjee AK, Chakraborty I (2018). Neurochemicals, behaviours and psychiatric perspectives of neurological diseases. Neuropsychiatry (London).

[101] Klein TA, Ullsperger M, Danielmeier C (2013). Error awareness and the insula: Links to neurological and psychiatric diseases. Front Hum Neurosci.

[102] Zhu Y, Liu C, Pang Z (2019). Dendrimer-based drug delivery systems for brain targeting. Biomolecules.

[103] Lee JH, Jung H Il (2013). Biochip technology for monitoring posttraumatic stress disorder (PTSD). Biochip J.

[104] Verkhratsky A, Parpura V (2016). Astrogliopathology in neurological, neurodevelopmental and psychiatric disorders. Neurobiol Dis.

[105] Martínez-Arán A, Vieta E, Colom F, Reinares M, Benabarre A, Gastó C (2000). Cognitive dysfunctions in bipolar disorder: Evidence of neuropsychological disturbances. Psychother Psychosom.

[106] Rothman S (2018). Intrinsic gains and career commitment among formal caregivers of patients with a major neurocognitive disorder. Diss Abstr Int Sect B Sci Eng.

[107] Kendler KS (2016). The nature of psychiatric disorders. World Psychiatry.

[108] Doswald S, Stark WJ, Beck-Schimmer B (2019). Biochemical functionality of magnetic particles as nanosensors: how far away are we to implement them into clinical practice?. J Nanobiotechnology.

[109] White JJ, Arancillo M, Stay TL, George-Jones NA, Levy SL, Heck DH (2014). Cerebellar zonal patterning relies on purkinje cell neurotransmission. J Neurosci.

[110] Kurzweil R, Grossman T (2009). Fantastic voyage: Live long enough to live forever: The science behind radical life extension questions and answers. Stud Health Technol Inform.

[111] Zeng J, Yu W, Dong X, Zhao S, Wang Z (2019). A nanoencapsulation suspension biomimetic of milk structure for enhanced maternal and fetal absorptions of DHA to improve early brain development. Biol Med.

[112] Furtado D, Björnmalm M, Ayton S, Bush AI, Kempe K, Caruso F (2018). Overcoming the blood-brain barrier: the role of nanomaterials in treating neurological diseases. Adv Mater.

[113] Golden RN (2016). Disrupting the adverse interplay between psychiatric and medical illnesses. Psychosom Med.

[114] Ziegler M, Kohlstedt H (2013). Mimic synaptic behavior with a single floating gate transistor: A MemFlash synapse. J Appl Phys.

[115] Jayaraj RL, Azimullah S, Beiram R, Jalal FY, Rosenberg GA (2019). Neuroinflammation: friend and foe for ischemic stroke. J Neuroinflammation.

[116] Jilka SR, Scott G, Ham T, Pickering A, Bonnelle V, Braga RM (2014). Damage to the salience network and interactions with the default mode network. J Neurosci.

[117] Dixon ML, Andrews-Hanna JR, Spreng RN, Irving ZC (2017). Interactions between the default network and dorsal attention network vary across default subsystems, time, and cognitive states. Neuroimage.

[118] Provenza NR, Matteson ER, Allawala AB, Barrios-Anderson A, Sheth SA (2019). The case for adaptive neuromodulation to treat severe intractable mental disorders. Front Neurosci.

[119] Lallouette J, De Pittà M, Ben-Jacob E, Berry H (2014). Sparse short-distance connections enhance calcium wave propagation in a 3D model of astrocyte networks. Front Comput Neurosci.

[120] Fields RD, Stevens-Graham B (2002). Neuroscience: New insights into neuron-glia communication. Science.

[121] Takagi K (2020). Principles of mutual information maximization and energy minimization affect the activation patterns of large scale networks in the brain. Front Comput Neurosci.

[122] Modi G, Pillay V, Choonara YE (2010). Advances in the treatment of neurodegenerative disorders employing nanotechnology. Ann N Y Acad Sci.

[123] Smith ES, Porterfield JE, Kannan RM (2019). Leveraging the interplay of nanotechnology and neuroscience: Designing new avenues for treating central nervous system disorders. Adv Drug Deliv Rev.

[124] Lal R (2017). Smart nano-shuttles for on-demand and targeted personalized medicine. J Biotechnol.

[125] Koczera P, Liu Z, Gremse F, Kiessling F, Lammers T (2012). USPIO-containing PBCA microbubbles for mediating and monitoring blood-brain barrier permeation. Mol Imaging Biol.

[126] Chhikara BS, Misra SK, Bhattacharya S (2012). CNT loading into cationic cholesterol suspensions show improved DNA binding and serum stability and ability to internalize into cancer cells. Nanotechnology.

[127] Chhikara BS, Parang K (2010). Development of cytarabine prodrugs and delivery systems for leukemia treatment. Expert Opin Drug Deliv.

[128] Habibi N, Quevedo DF, Gregory J V, Lahann J (2020). Emerging methods in therapeutics using multifunctional nanoparticles. WIREs Nanomed Nanobiotech.

[129] Yi YW, Lee JH, Kim S-Y, Pack C-G, Ha DH (2020). Advances in analysis of biodistribution of exosomes by molecular imaging. Int J Mol Sci.

[130] Samal J, Rebelo AL, Pandit A (2019). A window into the brain: Tools to assess pre-clinical efficacy of biomaterials-based therapies on central nervous system disorders. Adv Drug Deliv Rev.

[131] Choudhury FK, Rivero RM, Blumwald E, Mittler R (2017). Reactive oxygen species, abiotic stress and stress combination. Plant J.

[132] Zhou X, Hao Y, Yuan L, Pradhan S, Shrestha K (2018). Nano-formulations for transdermal drug delivery: A review. Chinese Chem Lett.

[133] Khan AR, Yang X, Fu M, Zhai G (2018). Recent progress of drug nanoformulations targeting to brain. J Control Release.

[134] Strafella C, Caputo V, Galota MR, Zampatti S, Marella G (2018). Application of Precision Medicine in Neurodegenerative Diseases. Front Neurol.

[135] Luan X, Sansanaphongpricha K, Myers I, Chen H, Yuan H, Sun D (2017). Engineering exosomes as refined biological nanoplatforms for drug delivery. Acta Pharmacol Sin.

[136] Duan M, Shapter JG, Qi W, Yang S, Gao G (2018). Recent progress in magnetic nanoparticles: synthesis, properties, and applications. Nanotechnology.

[137] Jia T, Rao J, Zou L, Zhao S, Yi Z, Wu B (2018). Nanoparticle-encapsulated curcumin inhibits diabetic neuropathic pain involving the P2Y12 receptor in the dorsal root ganglia. Front Neurosci.

[138] Devasena Umai R, Brindha Devi P, Thiruchelvi R (2018). A review on dna nanobots - A new technique for cancer treatment. Asian J Pharm Clin Res.

[139] Sacco R, Cacci E, Novarino G (2018). Neural stem cells in neuropsychiatric disorders. Curr Opin Neurobiol.

[140] David AS, Bedford N, Wiffen B, Gilleen J (2012). Failures of metacognition and lack of insight in neuropsychiatric disorders. Philos Trans R Soc B Biol Sci.

[141] Thirioux B, Harika-Germaneau G, Langbour N, Jaafari N (2020). The relation between empathy and insight in psychiatric disorders: phenomenological, etiological, and neuro-functional mechanisms. Front Psychiatry.

[142] Mondragón JD, Maurits NM, De Deyn PP (2019). Functional neural correlates of anosognosia in mild cognitive impairment and alzheimer's disease: a systematic review. Neuropsychol Rev.

[143] Reddy MS (2016). Lack of insight in psychiatric illness: A critical appraisal. Ind J Psychol Med.

[144] Riggs SE, Grant PM, Perivoliotis D, Beck AT (2012). Assessment of cognitive insight: A qualitative review. Schizophr Bull.

[145] Linden M, Godemann F (2007). The differentiation between “lack of insight” and “dysfunctional health beliefs” in schizophrenia. Psychopathology.

[146] Vannini P, Hanseeuw B, Munro CE, Amariglio RE, Marshall GA, Rentz DM (2017). Anosognosia for memory deficits in mild cognitive impairment: Insight into the neural mechanism using functional and molecular imaging. NeuroImage Clin.

[147] Veiga-Fernandes H, Artis D (2018). Neuronal-immune system cross-talk in homeostasis. Science.

[148] Wu Y, Briley K, Tao X (2016). Nanoparticle-based imaging of inflammatory bowel disease. Wiley Interdiscip Rev Nanomed Nanobiotech.

[149] Chhikara BS, Kumar R, Rathi B, Krishnamoorthy S, Kumar A (2016). Prospects of applied nanomedicine: potential clinical and (bio)medical interventions via nanoscale research advances. J Mater Nanosci.

[150] Chhikara BS (2017). Current trends in nanomedicine and nanobiotechnology research. J Mater Nanosci.

[151] Nguyen TA, Assadi AA (2018). Smart Nanocontainers: Preparation, Loading/Release Processes and Applications. Kenkyu J Nanotechnol Nanosci.

[152] Mircioiu C, Voicu V, Anuta V, Tudose A, Celia C, Paolino D (2019). Mathematical modeling of release kinetics from supramolecular drug delivery systems. Pharmaceutics.

[153] Abbott NJ, Patabendige AAK, Dolman DEM, Yusof SR, Begley DJ (2010). Structure and function of the blood-brain barrier. Neurobiol Dis.

[154] Garabadu D, Agrawal N, Sharma A, Sharma S (2019). Mitochondrial metabolism: A common link between neuroinflammation and neurodegeneration. Behav Pharmacol.

[155] Lengyel M, Kállai-Szabó N, Antal V, Laki AJ, Antal I (2019). Microparticles, microspheres, and microcapsules for advanced drug delivery. Sci Pharm.

